# Genotypic and molecular characterization of a moderately thermophilic cyanobacterium, *Gloeocapsa* sp. strain BRSZ

**DOI:** 10.1016/j.engmic.2025.100226

**Published:** 2025-08-05

**Authors:** Sasiprapa Samsri, Tanwalee Deprom, Chananwat Kortheerakul, Sophon Sirisattha, Stephen B. Pointing, Hakuto Kageyama, Rungaroon Waditee-Sirisattha

**Affiliations:** a Department of Microbiology, Faculty of Science, Chulalongkorn University, Phayathai Road, Pathumwan, Bangkok 10330, Thailand; b Thailand Institute of Scientific and Technological Research (TISTR), Khlong Luang, Pathum Thani 12120, Thailand; c Department of Biological Sciences, National University of Singapore, Singapore 117557, Singapore; d Department of Chemistry, Faculty of Science and Technology, Meijo University, 1-501 Shiogamaguchi, Tenpaku-ku, Nagoya, Aichi 468-8502, Japan; e Graduate School of Environmental and Human Sciences, Meijo University, 1-501 Shiogamaguchi, Tenpaku-ku, Nagoya, Aichi 468-8502, Japan

**Keywords:** Polyphasic taxonomy, Unicellular cyanobacteria, *Gloeocapsa*, Extremophile, Thermophile, Heterotrophy, Hot spring

## Abstract

A unicellular-colonial cyanobacterium, designated “BRSZ,” was isolated from a neutral-alkaline hot spring in Thailand. Morphological characterization revealed distinctive features consistent with those of the genus *Gloeocapsa*. Physiological assessments demonstrated that BRSZ is a moderately thermophilic and halotolerant cyanobacterium with the potential for chemoheterotrophic growth in dark conditions. Molecular phylogenetic analysis based on 16S ribosomal RNA (rRNA) gene sequences placed BRSZ within a well-defined *Gloeocapsa* clade, a finding corroborated by 16S–23S internal transcribed spacer (ITS) rRNA secondary structure analyses. Genome comparisons, including average nucleotide identity (ANI), genome-to-genome distance (GGD), and digital DNA-DNA hybridization (dDDH), between strain BRSZ and closely related taxa showed an ANI value of 95.45 %, near the lower boundary of the species delineation threshold (95–96 %). A GGD of 0.0374 (>0.0258) and dDDH of 69 % (<70 %) further supported genomic differentiation. Genome-based analysis revealed a mycosporine-like amino acid biosynthetic gene cluster likely involved in sunscreen compound production. Cultivation-based production of a UV-absorbing compound confirmed the functional relevance of this gene cluster. These findings expand the described diversity within the *Gloeocapsa* complex and enhance our understanding of the taxonomy of this group. In addition, they underscored the importance of hot spring environments as sources of novel extremophiles.

## Graphical abstract

**Figure:**
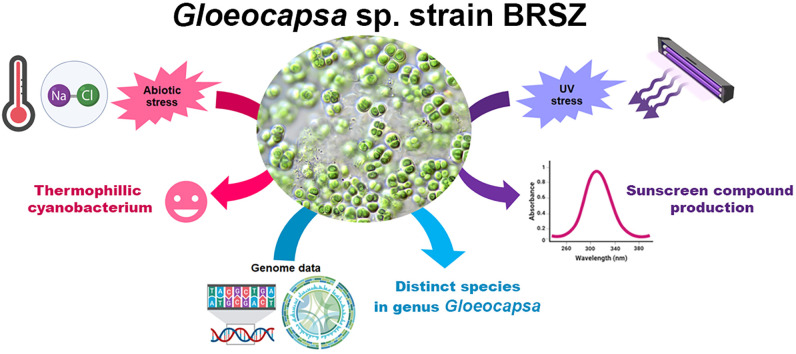

## 1 Introduction

Extremophiles are organisms capable of thriving under harsh physical and chemical conditions and are of great scientific interest. These organisms can endure extreme environments, which highlights their ecological significance. By adapting to such adverse conditions, extremophiles extend the known physical and chemical boundaries of life on Earth, providing crucial models for understanding molecular evolution, distinct physiological processes, and potential biotechnological applications [1]. According to fossils dating back 3.5 billion years, cyanobacteria comprise an enormously diverse group of prokaryotes that inhabited Earth [2]. Cyanobacteria have become highly adaptive over time. They thrive in extreme environments encompassing a vast range of terrains, such as hot springs, hypersaline lakes, deserts, volcanoes, and polar regions [[3], [4], [5], [6]]. Among these extreme environments, high temperatures present a unique opportunity to investigate the specific adaptations and survival mechanisms of cyanobacteria**.** Various ecological niches such as hot springs, volcanic sites, and other extreme-temperature areas serve as habitats for thermophiles [7]. Surprisingly, cyanobacteria exhibit progressive growth at high temperatures. In ecological niches such as hot springs, the temperature can reach up to 73 °C [8], the upper limit for photosynthesis. In hot springs, cyanobacteria are associated with microbial biofilms and are the dominant primary producers [9]. At higher temperatures, chemoautotrophic biofilms become predominant in neutral-alkaline springs [10], whereas in acidic hot springs, the photosynthetic microbiota may be dominated by eukaryotic algae [11]. Hot springs harbor a variety of microbial communities, including planktonic, sedimentary, and biofilm populations, making them valuable systems for studying microbial ecology. Investigations of hot spring sediments and planktonic habitats have shown that the assembly of local communities is highly influenced by abiotic factors, such as temperature and pH [12,13]. Photosynthetic biofilms represent a critical microbial niche in hot springs, forming the dominant biomass in neutral-alkaline springs at temperatures ranging from the onset of thermophily at 40–45 °C to the upper limits for photosynthesis at 73 °C [8]. These neutral-alkaline hot springs are widely found in tectonic landscapes worldwide. Cyanobacteria-dominated biofilms have been described from hot springs in many regions including Africa [14], China [15], Europe [16,17], India [18], Japan [19], Australasia [20,21], North America [22,23], South America [24], and Southeast Asia [25]. Previous research has indicated that Southeast Asian hot springs support a diverse range of cyanobacteria and may also be a source of novel phylogenetic lineages and potential new species awaiting description. In Thailand, neutral-alkaline hot springs are widely distributed [26], and several descriptive studies have identified cyanobacteria in hot springs, including *Calothrix, Cyanothece, Fischerella, Phormidium, Pleurocapsa, Oscillatoria*, and *Synechococcus* from northern Thailand [27,28], and *Mastigocladus* (*Fischerella*) from southern Thailand [29]. A commonly encountered cyanobacterial genus includes *Gloeocapsa*. This genus and closely related taxa have also been reported in other hot springs and diverse extreme environments, such as *Gloeocapsa* sp. PCC 7428 [30], *Gloeocapsa gelatinosa* Kützing [31], *Gloeocapsa* sp. HG2 [32], *Gloeocapsopsis dulcis*, and *Gloeocapsopsis diffluens* [33]. This observation suggests that this genus may exhibit characteristics of a polyextremophile that adapts to multiple environmental stresses.

We hypothesized that exploring hot springs in Thailand, which are currently underrepresented in the global effort to describe hot spring cyanobacteria, would potentially yield novel *Gloeocapsa* isolates and, in turn, lead to an improved understanding of the ecology, adaptive mechanisms, and molecular evolution of extremophiles. The accurate identification and assessment of newly isolated cyanobacterial strains are essential for taxonomic characterization and contribute to cyanobacterial systematics. The taxonomic scheme for cyanobacteria can be resolved using a combination of methods (e.g., collecting genotypic, chemotaxonomic, and phenotypic data to determine taxonomic positions). This combination is referred to as the polyphasic approach. Genetic information, such as 16S ribosomal RNA (rRNA) gene data, is the primary method of evaluation. This can be supplemented by secondary criteria, including morphology, specific biochemical components, and ecophysiology. Combining these methods provides a modern, unique, and comprehensive approach to taxonomic classification. Therefore, a polyphasic approach is currently the most popular method for classifying microbes, including cyanobacteria [[34], [35], [36]]. In this work, we present the results of isolation and polyphasic assessment of a new thermophilic and halotolerant cyanobacterial strain of *Gloeocapsa* sp. strain Bentong-Raub Suture Zone (BRSZ), which was sampled from a neutral-alkaline hot spring at Bo Khlueng, Ratchaburi Province, Thailand. Our approach integrates morphological observations, molecular analyses, and chemotaxonomic profiling. These findings enhance our understanding of the taxonomy of the *Gloeocapsa* complex and underscore the importance of conducting additional floristic surveys of polyextremophiles in tropical coastal environments that have been inadequately studied.

## 2 Materials and methods

### 2.1 Environmental sampling and isolation of cyanobacteria

Photosynthetic microbial biofilms were aseptically sampled at a thermally neutral-alkaline hot spring site (Bo Khlueng: 13.7368 °N, 99.2395 °E, Ratchaburi Province) in central Thailand in August 2022. Abiotic variables (temperature, pH, electrical conductivity [EC], hydrogen sulfide level, total alkalinity, and phosphate, nitrate, and nitrite content) were measured on-site using handheld probes and colorimetric test kits, as previously described [37]. Temperature was measured using a portable thermometer (Esco, Singapore). The EC was measured using a conductivity meter (Hach, USA). Total alkalinity, pH, phosphate, nitrate, and nitrite were measured using a Hach Water Quality Kit (Hach) and an SJ 16-in-1 Water Kit (SJ 16-in-1 Water, China). Hydrogen sulfide levels were measured using kits from Hach and ITS (Germany). The BRSZ strain was isolated and cultivated using standard methods. First, photosynthetic microbial biofilms were serially diluted in BG11 liquid medium [30], followed by micromanipulation to isolate single BRSZ cells. For micromanipulation, an inverted microscope (Leica DMi1 Inverted Microscope, Leica Microsystems [SEA], Singapore) equipped with a micromanipulator and sterile glass micro-capillaries (customized with an inner diameter of 25–50 μm) was used to capture a single BRSZ cell from a culture suspension into 200 μl of sterile BG11 liquid medium. The single cell in BG11 liquid medium was illuminated with cool white luminescent lamps providing an average irradiation of 50 μmol photons m^−2^ ⋅ s^−1^. After observing the presence of a light-green culture (typically 7–14 days), the culture was plated onto BG11 agar to obtain single colonies. A single BRSZ colony was confirmed as an axenic culture. This strain was deposited at the TISTR Culture Collection Center (https://www.tistr.or.th/tistr_culture/index.php) under the TISTR number 9501. Routine subculture was then performed, with a BRSZ culture in 300 mL Erlenmeyer flasks containing 100 mL of BG11 liquid medium, placed in an orbital shaker at room temperature (30 ± 1 °C) with cool white luminescent lamps providing an average irradiation of 50 μmol photons m^−2^ ⋅ s^−1^. All cultures were grown at 30 ± 1 °C unless otherwise specified.

### 2.2 Microscopy and staining

The morphologies of the cyanobacterial strains were investigated at 100 × magnification using a light microscope (Nikon Upright Microscope Eclipse Ni-U, Japan) equipped with an image acquisition system (NIS Elements D, Japan). Gram staining was performed according to the standard protocol [38].

### 2.3 Effect of heterotrophic growth and stress treatments on BRSZ growth

Intensive cultivation of BRSZ was carried out in glass vessels (Erlenmeyer flasks) containing 100 mL of BG11 medium. Heterotrophy of BRSZ was tested by transferring the inoculum from liquid BG11 medium to BG11 agar supplemented with glucose as a carbon source and incubating under dark conditions for two months. For stress treatments, different types of abiotic stresses (e.g., fluctuating, thermal, osmotic, and UV stresses) were applied. To investigate temperature-dependent growth, the BRSZ cultures were incubated at five different temperatures: 15 ± 1, 30 ± 1, 40 ± 1, 55 ± 1, and 60 ± 1 °C. The OD_730_ was measured to observe culture growth after 48 h of continuous illumination at 50 μmol photons m^−2^ ⋅ s^−1^.

A salt upshock experiment was also performed to test salt tolerance. Ten milliliters of the BRSZ culture in BG11 liquid medium with OD_730_ ≈ 0.6–0.8 was harvested and transferred into an equal volume of fresh BG11 medium containing NaCl at various concentrations (0–1.5 M NaCl). The cells were photoautotrophically cultured in 25 mL Erlenmeyer flasks in an orbital shaker for 3 days. In addition to the liquid culture experiment, salt tolerance was assessed by inoculating BG11 agar plates supplemented with NaCl (0–1.5 M). The plates were incubated under standard photoautotrophic conditions, and cell survival and growth were monitored over 30 days. For UV stress, strain growth was investigated using 2-month-old cells cultured on BG11 agar. The strain was exposed to continuous UVA (365 nm) fluorescent light for 3, 6, or 12 h. All growth experiments were repeated at least three times.

### 2.4 Genomic DNA preparation and ITS rRNA sequencing

Total genomic DNA (gDNA) was isolated from a 50 mL culture of cyanobacterial BRSZ cells grown in liquid BG11 medium. Cells were collected via centrifugation and subjected to gDNA isolation using a DNeasy® PowerLyzer® PowerSoil® Kit (Qiagen, Germany), following the manufacturer’s instructions. The extracted gDNA was quantified using a Nanodrop 2000 UV–Vis spectrophotometer (Thermo Fisher Scientific, USA). Taxonomic diversity was estimated using the 16S rRNA gene and the 16S–23S rRNA ITS regions. The molecular marker genes were amplified using polymerase chain reaction (PCR) in a Thermal Cycler (Model C-1000 Touch™, Bio-Rad Laboratories, USA) with the following reaction components: 50 ng of gDNA, 0.2 µM of each primer (forward and reverse), 200 µM of dNTPs, 1 × Standard *Taq* Buffer, and 1 U *Taq* DNA polymerase (New England Biolabs, USA); the total reaction volume was 25 µL. The primer sequences for the 16S rRNA gene were 5′-AGAGTTTGATCCTGGCTCA-3′ (Forward) and 5′-CTAAGGTGATCCAGCCACA-3′ (Reverse). For ITS region amplification, the primer set used was 5′-TGTACACACCGCCCGTC-3′ (Primer 322, Forward) and 5′-CTCTGTGTGCCTAGGTATCC-3′ (Primer 340, Reverse), as described previously [39]. The PCR products were sequenced using the primers employed in the PCR conditions by Macrogen, Inc. (Korea). The 16S rRNA gene and ITS sequences were deposited in GenBank under accession numbers PP907067 and PQ130443, respectively [40].

### 2.5 Phylogenetic reconstruction of the 16S rRNA gene

The 16S rRNA gene was used for molecular phylogenetic analysis. The dataset for this analysis comprised cyanobacterial sequences identified through a BLAST search (https://blast.ncbi.nlm.nih.gov/Blast.cgi) that were closely related to those obtained from the BRSZ strain. The phylogenetic analysis used 94 taxa for the 16S rRNA gene (Supplementary Table S1), which are available in GenBank (http://www.ncbi.nlm.nih.gov/GenBank/; accessed May 2024). *Gloeobacter violaceus* PCC 7421 was used as the outgroup. The evolutionary relationships of the 16S rRNA genes were reconstructed using MEGA 11 [41] with the neighbor-joining method [42] and bootstrap consensus trees based on 1000 replicates [43]. Evolutionary distances were calculated using the maximum composite likelihood method [44] and are represented as the number of base substitutions per site.

### 2.6 Procedures for whole-genome sequence comparison

To assess genomic similarity, the average nucleotide identity by BLAST (ANIb) was calculated using JSpeciesWS v4.2.3 (https://jspecies.ribohost.com/jspeciesws/#analyse) [45]. The whole-genome sequences of strain BRSZ [40] (manually retrieved from accession numbers NZ_JBEGHC000000001.1–NZ_JBEGHC000000042.1) and related taxa (*Gloeocapsa* sp. PCC7428; accession number NC_019745.1) in FASTA format were uploaded, and ANIb values were determined by aligning homologous regions and measuring nucleotide similarity. The average nucleotide identity (ANI) threshold for species delineation is 95–96 %, with lower values suggesting distinct species [46]. The genome-to-genome distance (GGD) was calculated using the Genome-to-Genome Distance Calculator v3.0 (http://ggdc.dsmz.de) [47]. The GGD and digital DNA-DNA hybridization (dDDH) values were obtained by uploading the whole-genome sequences of the two strains. The species cutoff was 0.0258 for GGD and 70 % for dDDH, with values above the GGD cutoff and below the dDDH cutoff, respectively, suggesting distinct species [48]. Biosynthetic gene clusters encoding secondary metabolites were predicted and annotated using antiSMASH software (version 7.1.0) [49].

### 2.7 Prediction of secondary structures

The 16S–23S rRNA ITS sequences of strain BRSZ and nine related taxa were aligned using CLUSTALW (https://www.genome.jp/tools-bin/clustalw). Conserved domains within the 16S–23S ITS region, including D1-D1′, D2, D3, tRNA^Ile^, and D4, as well as variable regions such as V2, Box B, V3, and D5, were identified as described previously [39]. The secondary structures of the entire ITS region and identified fragments were predicted individually using the RNA folding form on the UNAfold web server (http://www.unafold.org/mfold/applications/rna-folding-form.php) with default settings [50]**.** Secondary structure predictions were performed based on the minimum free energy under the default conditions of the server.

### 2.8 Extraction of UV-absorbing compounds under UV treatment

Cells of the strain BRSZ cultured on BG11 agar plates and exposed to UV light, as described in the stress treatment section, were used to extract UV-absorbing compounds. The extraction process followed a previously described protocol [51], with modifications. Typically, 0.8 mL of methanol was added to 0.1 g of the fresh cell weight. The cells were then disrupted using a sonicator (Vibra-Cell™ Ultrasonic Liquid Processors VCX-130, Sonics, USA) with a program of 30-s pulse on, 10-s pulse off, for a total of 10 min on, ensuring complete disruption. After sonication, the mixture was centrifuged, and the supernatant was dried using a Centrifugal Vacuum Concentrator (Eppendorf, Germany). The dried material was then dissolved in Milli-Q water (0.5 mL), and any undissolved compounds were removed via further centrifugation. To eliminate unassociated pigments, 0.01 mL of chloroform was added, followed by centrifugation. The supernatant (aqueous phase) was transferred to an Amicon Ultra-4 Ultracel-3 K Centrifugal Filter (Merck, Germany) and centrifuged until most of the solution passed through the membrane, and the flow-through fraction was collected. The UV–Vis spectrum of the resulting sample containing the aqueous phase of the UV-absorbing compound mixture was measured using a UV–Vis spectrophotometer (BioMate 3S Spectrophotometer, Thermo Scientific, USA).

## 3 Results and discussion

### 3.1 Morphological characterization of the cyanobacterial strain BRSZ

**Fig. 1:**
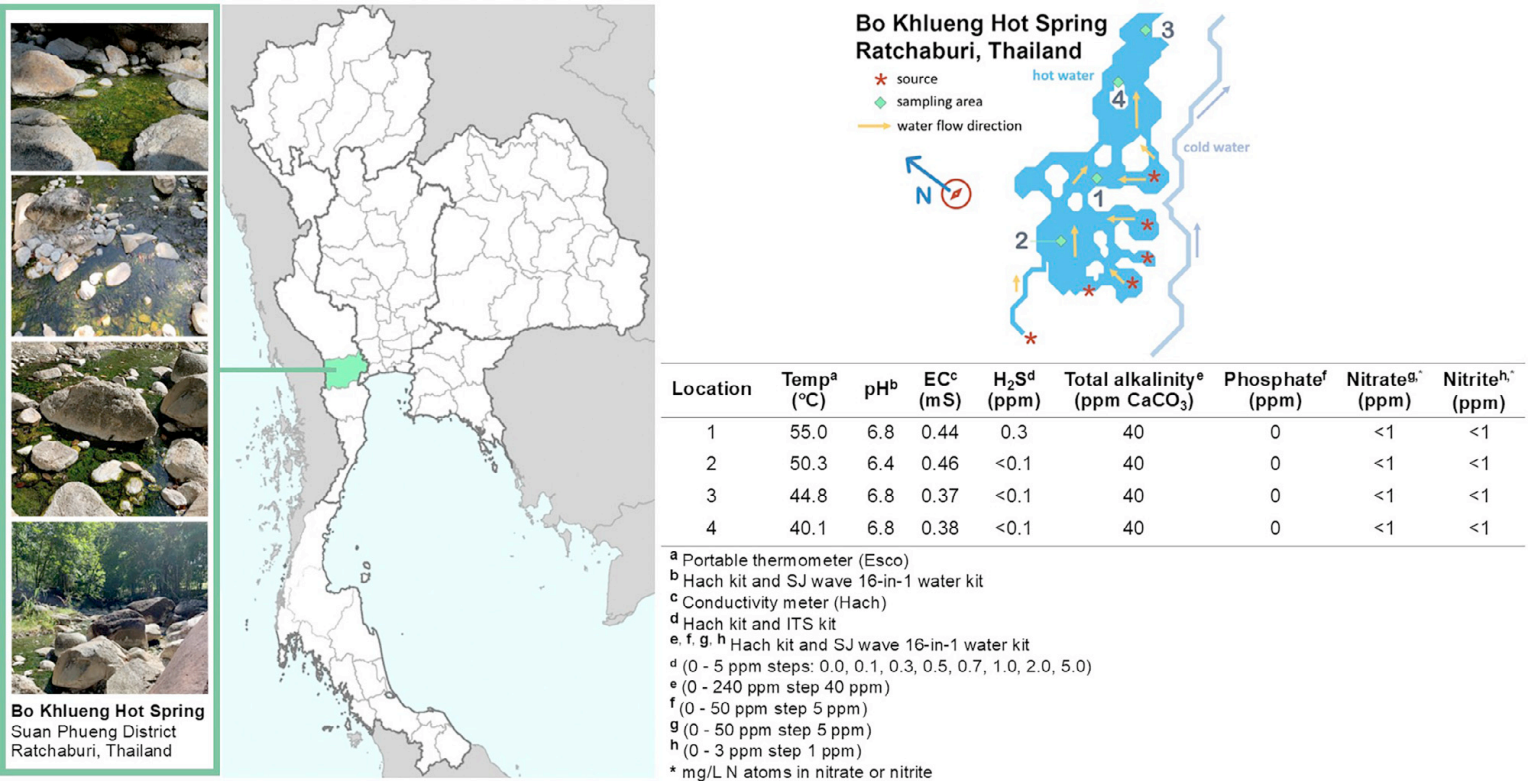
**Photographs and map of the sampling location at Bo Khlueng Hot Spring, Suan Phueng District, Ratchaburi, Thailand.** Shown is a diagram depicting the sampling area around Bo Khlueng Hot Spring. The temperatures of locations 1 to 4 are approximately 55, 50, 45, and 40 °C, respectively. Abiotic variables (temperature, pH, electrical conductivity [EC], hydrogen sulfide, total alkalinity, phosphate, nitrate, and nitrite) were measured on-site using handheld probes and colorimetric test kits (Hach, USA; SJ 16-in-1 water, China, and ITS, USA).

The neutral-alkaline hot spring site at Bo Khlueng is a natural hot spring that is not affected by nearby human activity. Physical parameters were measured at temperatures of 40, 45, 50, and 55 °C, with the maximum temperature being 55 °C (Fig. 1). At the locations depicted in Fig. 1, we aimed to isolate cyanobacteria from the site with the highest temperature. Photosynthetic microbial films collected from location 1 were purified to obtain axenic cultures. At this location, at least eight cyanobacterial strains were isolated (Supplementary Figure S1). Each axenic culture was subjected to various abiotic stressors. Notably, BRSZ was among the eight strains that exhibited robust tolerance to high temperature, high salt concentration, and UV treatment (see the next section for details). Therefore, we focused on this strain in the present study.

**Fig. 2:**
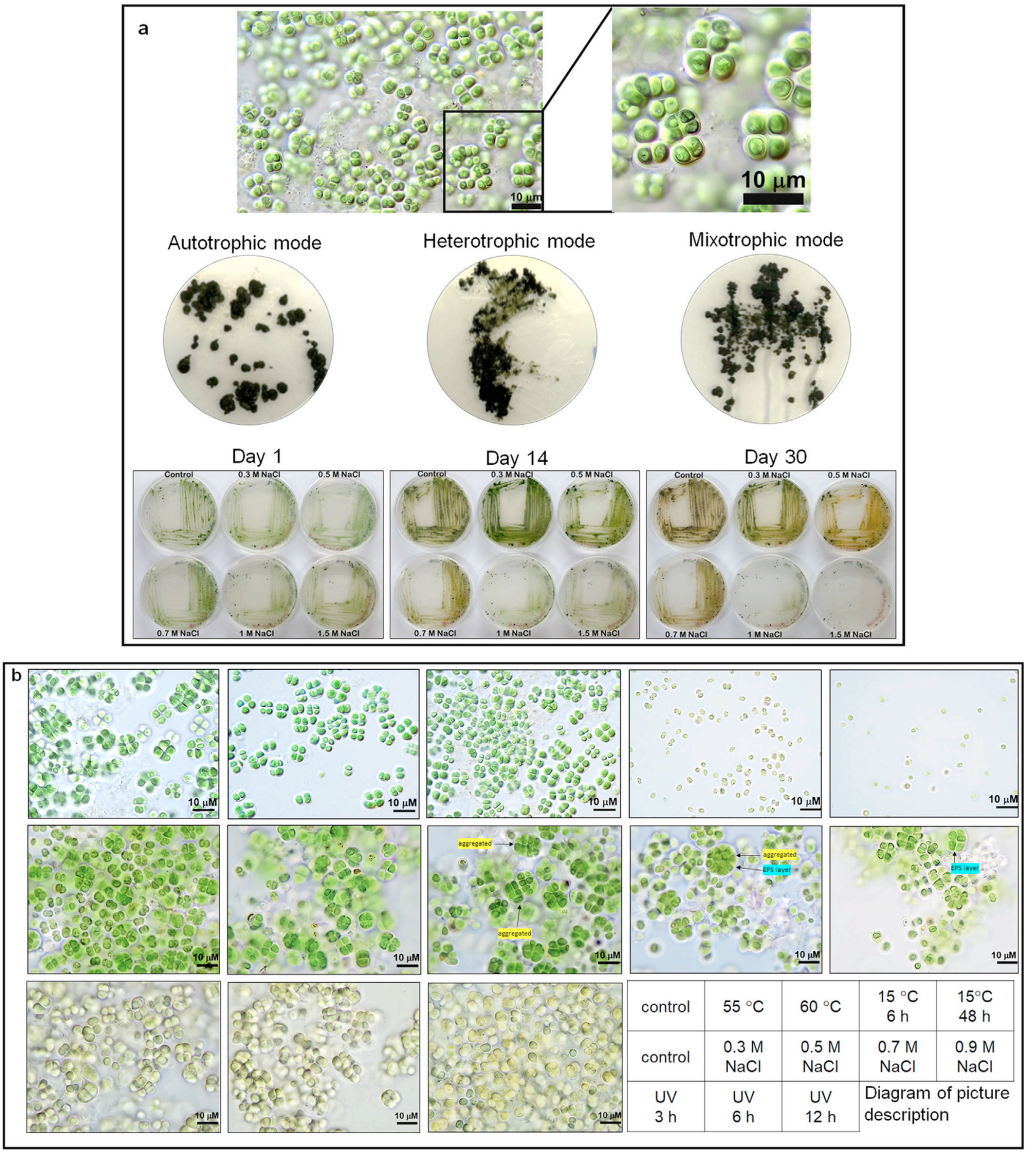
**Photomicrographs and observation of the growth of the cyanobacteria strain BRSZ.** (a) Micrographs were recorded in differential interference contrast mode at 100 × magnification. Strain BRSZ can grow under different culture conditions: autotrophic (BG11 agar with continuous light), heterotrophic (BG11 agar supplemented with 15 g ⋅ L^-1^ glucose under dark conditions), mixotrophic (BG11 agar supplemented with 15 g ⋅ L^-1^ glucose with continuous light), and salt (BG11 agar supplemented with NaCl 0−1.5 M) for 1, 14, and 30 days. (b) Strain BRSZ can grow under fluctuating temperatures, high salt, and UV stresses. The arrows indicate the aggregation of cells and the extracellular polymeric substance (EPS) layer with increasing NaCl concentrations.

When maintained on BG11 agar plates, BRSZ cells formed thick, dense, green colonies (Fig. 2a). Microscopic observations revealed spherical colonies containing small cell groups resulting from binary fission held together by mucilage. Individual cells and colony sizes ranged from approximately 2.6–3.4 µm and 4.0–9.9 µm, respectively (Fig. 2a). The strain BRSZ resembles the genus *Gloeocapsa* based on its morphological characteristics. According to Bergey’s manual [52], *Gloeocapsa* can be unicellular or composed of small groups of cells that divide via binary fission in two or more planes at right angles, forming aggregates held together by a concentric mucilage envelope. *Gloeocapsa* colonies are typically spherical, microscopic, and enclosed within larger mucilaginous masses, with cell sizes ranging from 3 to 10 µm in diameter [24].

The morphology of strain BRSZ closely resembles that of the genus *Gloeocapsa* but can also be confused with other common unicellular cyanobacteria, such as *Gloeocapsopsis* [33]. This similarity often leads to confusion in taxonomic classification. Therefore, a polyphasic approach was employed, utilizing additional analyses beyond morphology to achieve accurate classification.

### 3.2 Heterotrophy and survival of strain BRSZ under extreme environmental conditions

Cyanobacteria are photosynthetic autotrophs; however, several reports have demonstrated the heterotrophic growth of several strains of cyanobacteria on organic compounds such as sugars [53]. We then tested whether BRSZ was capable of chemoheterotrophic growth using glucose as the carbon source. As shown in Fig. 2a, the cultures were incubated for two months. They displayed growth capabilities in three different modes: autotrophic, heterotrophic, and mixotrophic. BRSZ can grow in heterotrophic conditions under 15 g ⋅ L^-1^ of glucose (Fig. 2a). This strain was demonstrated to be a mixotroph and a photoheterotroph (Supplementary Figure S2). The highest specific growth rate (µ) was observed under photoheterotrophic conditions (µ = 0.43 ± 0.01 day^-1^) (Supplementary Figure S2), confirming the strain’s ability to utilize light as an energy source and glucose as an organic carbon source. Additionally, when cultured with light, glucose, and bicarbonate, the specific growth rate was 0.35 ± 0.02 day^-1^ (Supplementary Figure S2). These results suggest that BRSZ prefers photoautotrophy, perhaps to avoid photoinhibition, even when the specific growth rate is low. Heterotrophy can be beneficial in certain contexts, such as increasing biomass yield. The *Gloeocapsa* sp. PCC 7428 and *Gloeocapsa* sp. PCC 7501 could grow photoheterotrophically on carbon sources such as glucose [30,53]. In contrast, *Gloeocapsopsis* is primarily known for its ability to thrive under low water availability conditions, such as the Atacama Desert and maritime Antarctica; however, there is no direct evidence in the provided contexts to suggest its growth under heterotrophic conditions [33,54,55]. Thus, strain BRSZ is likely a taxon within the genus *Gloeocapsa*.

BRSZ was subjected to various stress treatments to evaluate its tolerance and morphological adaptability (Fig. 2b). Because of its origin in high-temperature habitats, temperature-dependent growth characteristics were observed (first row in Fig. 2b). The results showed that the morphology of strain BRSZ at room temperature (30 ± 1 °C) was similar to that at 55 ± 1 °C, with cells exhibiting the same size and stage of cell division (approximately four cells). However, at 60 ± 1 °C, the cells were smaller. To characterize it as a thermophile, the growth rate of BRSZ was assessed at various temperatures. The optimal temperature of the strain BRSZ is 40 °C (specific growth rate = 0.29 ± 0.01 day^-1^) (Supplementary Figure S3). When subjected to low temperatures (15 ± 1 °C), the cells almost died, appearing very small, ceasing division, and exhibiting a yellowish color. These results suggest that the preferred temperature range for growth is between 30 and 50 °C; therefore, we concluded that strain BRSZ is a moderately thermophilic cyanobacterium. *Gloeocapsa* spp. have been reported in various thermal springs with a temperature range between 45 and 60 °C [14,28,31,56].

Additionally, BRSZ demonstrated remarkable salt tolerance (Fig. 2a and second row in Fig. 2b). The strains were maintained on BG11 agar plates supplemented with various NaCl (0–1.5 M) for 30 days. The results demonstrated that BRSZ could tolerate salt stress and survive in NaCl concentrations up to 0.7 M. However, at higher concentrations (1 M and 1.5 M NaCl), the cell population declined and eventually disappeared over the observation period (Fig. 2a). This suggests that BRSZ has a salt tolerance of up to 0.7 M NaCl, the highest tested concentration at which survival was sustained over the 30-day observation period. In the case of salt stress, the strain BRSZ can grow under high-salt conditions up to 1.5 M; however, its optimal growth is maximal under BG11 (no extra salt added), suggesting the BRSZ strain is a halotolerant strain rather than a halophilic strain (Supplementary Figure S3). Microscopic images revealed changes in the extracellular polymeric substance (EPS) layer, which enhances the cell’s ability to survive and function by exhibiting palmelloid formation (cell aggregation) in the EPS matrix with increasing NaCl concentration. Additionally, an increase in the intensity of the brown-yellow color in the EPS layer of cells was observed with increasing NaCl concentrations (second row in Fig. 2b). *Gloeocapsa* sp. N107 is capable of tolerating salt up to 2.2 M NaCl [57]. Other characterized *Gloeocapsa* strains, such as *Gloeocapsa* sp. UAM572 and *Gloeocapsa* sp. HG2, could grow at maximal salt concentrations of 0.34 M and 0.04 M NaCl, respectively [58]. This implies that *Gloeocapsa* can grow in various salinity levels.

After UV treatment, BRSZ cells maintained the same size as the control in terms of individual cells; however, cell division was inhibited. The cells appeared yellowish, suggesting that photosynthetic pigments, such as chlorophyll, were impaired and/or that other pigments were overproduced in response to UV stress. Overall, BRSZ demonstrated resilience to various environmental stressors, including different temperature regimes, salt stress, and UV radiation, suggesting that strain BRSZ is a polyextremophile. These stressors caused noticeable changes in both the morphology and cell division patterns of the strain under extreme conditions.

### 3.3 16S rRNA phylogeny

**Fig. 3:**
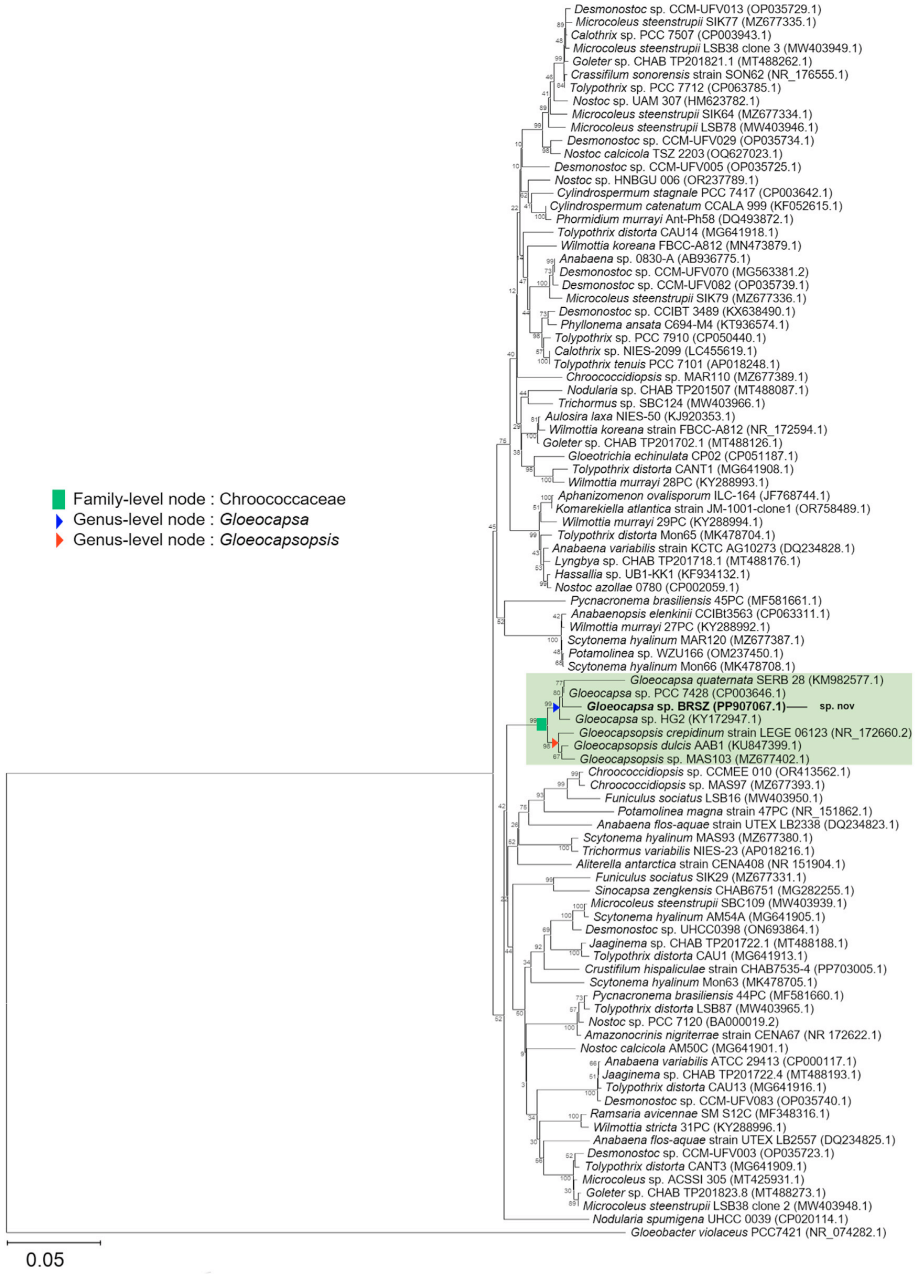
**Neighbor-joining phylogenetic tree of the 16S rRNA gene reconstructed from 94 nucleotide sequences.** The percentage of replicate trees in which the associated taxa clustered together in the bootstrap test (1000 replicates) is shown next to the branches. Strain BRSZ, in bold, represents the strain identified in this study. The tree is drawn to scale, with 0.05 substitutions per nucleotide position. All taxon names are followed by accession numbers in parentheses. The green box represents the branch containing only cyanobacteria in the family Chroococcaceae (green square node), which includes the clade of the genus *Gloeocapsa* and *Gloeocapsopsis* (blue and red triangle).

A molecular phylogenetic tree of the 16S rRNA gene was constructed from 94 sequences, with *Gloeobacter* as the outgroup (Fig. 3). Strain BRSZ, together with six taxa in the family Chroococcaceae, formed a well-defined clade as a monophyletic group, and appeared in a distinct branch that was a sister taxon to the genus *Gloeocapsa* (Fig. 3). Sequence identity analysis (Supplementary Table S1) indicated that the nearest neighbor of BRSZ was *Gloeocapsa* sp. PCC 7428 (98.85 % identity) (Table 1a), a strain isolated from a hot spring in Sri Lanka (55 °C) [30], followed by *Gloeocapsa* sp. HG2 (98.17 % identity), isolated from a hot spring in Israel (55 °C), *Gloeocapsopsis* spp. (96.48–97.02 % identity), and *Gloeocapsa quaternata* SERB 28 (94.99 % identity). The other taxa exhibited 85.06–92.21 % identities of their 16S rRNA gene to that of strain BRSZ (Supplementary Table S1).

**Table 1a:** Comparative whole-genome and single-gene sequence analysis of the strain BRSZ and *Gloeocapsa* sp. PCC 7428.

Genome Region	Whole genome sequence			Single-gene sequence	
Parameters	ANIb	GGD	dDDH	*16S rRNA*	*16S-23S* ITS dissimilarity
**Value**	95.45 % (78.03 % aligned)	0.0374	69.00 %	98.85 %	11.90 %
Threshold to separate species*	<95–96 % [46]	>0.0258 [48]	<70 % [48]	<99 % [55]	>7 % [62]

⁎ The threshold values used for species delamination as presented in the references [46,48,55,62].

According to the recommended guidelines for the use of 16S rRNA gene sequencing for cyanobacterial classification [59,60], strains within the same genus should be placed in a monophyletic branch and have at least 95 % similarity in their 16S rRNA gene sequences [59]. BRSZ, with 98.85 % similarity with *Gloeocapsa* sp. PCC 7428, exceeded the 95 % threshold required for classification within the same genus. This indicates that BRSZ should be considered a part of the genus *Gloeocapsa*. Moreover, strains with similar morphology but belonging to a phylogenetically distinct clade should be classified into separate genera [60]. Our results showed that BRSZ formed a monophyletic clade with *Gloeocapsa* (Fig. 3). Thus, strain BRSZ belongs to the genus *Gloeocapsa*, despite any morphological similarities it may share with strains from other genera, such as *Gloeocapsopsis*. Additionally, the percentage similarity of the 16S rRNA sequences within the *Gloeocapsa* clade was used to distinguish cyanobacterial taxa at the species level. Initially, a 16S rRNA sequence similarity threshold of <98.8 % was used to differentiate the cyanobacterial taxa at the species level [61]. More recent guidelines have suggested a stricter threshold of <99 % for strains within *Nostocales* [56]. A similarity of 98.85 % between the strains BRSZ and *Gloeocapsa* sp. PCC 7428 was slightly above the initial 98.8 % threshold for distinguishing species but below the more stringent 99 % threshold. This suggests that strain BRSZ is a species distinct from *Gloeocapsa* sp. PCC 7428. To further confirm this classification, we analyzed the whole-genome sequence and secondary structure of the 16S–23S rRNA ITS and examined the production of secondary metabolites in subsequent experiments.

### 3.4 Whole-genome sequence analysis

The draft genome sequence of strain BRSZ [40] was used to analyze the ANI, GGD, and dDDH values. These analyses compared the whole genome of BRSZ with that of its closest relative, *Gloeocapsa* sp. PCC7428. The ANI, which measures the percentage similarity of nucleotide sequences in conserved genes between two genomes, was calculated using the ANIb method. The results showed an ANIb value of 95.45 %, with 78.03 % of the nucleotide regions aligned (Table 1). This ANI value falls within or slightly above the species delineation threshold range of 95–96 %, suggesting that BRSZ is likely part of the same species as *Gloeocapsa* sp. PCC7428. However, for a more definitive classification, additional evidence from the GGD and dDDH analyses was considered. The GGD was inferred using high-scoring segment pairs [62], which were then converted into dDDH values using a generalized linear model. The results indicated a GGD value of 0.0374 and a dDDH value of 69 %. These values suggest that the two genomes are closely related but not identical. Since the established species delineation thresholds were a GGD of <0.0258 and a dDDH value greater than 70 %, these findings indicate that strain BRSZ and *Gloeocapsa* sp. PCC7428 are closely related at the genus level, but do not belong to the same species.

The characteristics of the selected *Gloeocapsa* spp. and BRSZ strains were then compared (Table 1b). Additionally, the genome assembly metrics of *Gloeocapsa* BRSZ and two other strains, PCC 7428 and PCC 73106, were compared (Supplementary Table S4). Genome-based analysis revealed that *Gloeocapsa* BRSZ harbors specific genes responsible for abiotic stress responses, such as molecular chaperones, glutathione S-transferase, and osmoprotectant metabolic enzymes (Supplementary Table S5). These BRSZ-specific genes are homologous to those of other cyanobacteria, but not *Gloeocapsa* sp. PCC 7428. We also identified several genes encoding sugar transporters and carbohydrate metabolism, which are presumably associated with heterotrophy. Lastly, antiSMASH analysis predicted secondary metabolite biosynthetic gene clusters such as nostophycin, minutissamide, puwainaphycin, nostopeptolide, and anabaenopeptin (Supplementary Table S5). As these secondary metabolites belong to a wide variety of chemical classes, these data offer the potential for exploring the production of compounds with biological properties.

**Table 1b:** Comparative characteristics of selected *Gloeocapsa* sp. and the strain BRSZ.

Taxon	Accession number		Original isolated	Salt-tolerant (NaCl)	Optimal growth Temp. (°C)	Dark-growth ability	MAAs	Whole genome sequence analysis^a^			Specific gene analysis			Ref.
	Whole genome	*16S rRNA*						ANIb ( %)	GGD	dDDH ( %)	*16S rRNA* similarity ( %)^a^	*16S-23S* ITS dissimilarity ( %)^a^	BGC of MAAs b	
*Gloeocapsa* sp. strain BRSZ	JBEGHC000000000.1	PP907067.1	Hot spring (55 °C)	0.7 M	40	Photoheterotrophic	✓	–	–	–	–	–	*MysA-MysB-MysC*	This study
*Gloeocapsa* sp. PCC 7428	CP003646.1	Glo7428_R0050	Hot spring (55 °C)	NR	NR	Photoheterotrophic	NR	95.45 (78.03 % aligned)	0.0374	69.00	98.85	11.90	*MysA-MysB-MysC*	[30]
*Gloeocapsa* sp. PCC 73,106	NZ_ALVY00000000.1	AB039000.1	Sphagnum bog	NR	NR	Photoheterotrophic	NR	66.55 (18.60 % aligned)	0.2377	18.50	84.72	NR	NR	[30]
*Gloeocapsa* sp. HG2	NR	KY172947.1	Hot spring (44.5 °C)	0.04 M	40–45	Photoheterotrophic	NR	NR	NR	NR	98.17	NR	NR	[32]
*Gloeocapsa* sp. UAM572	NR	MW544038.1	Desert	0.34 M	25	Photoheterotrophic	NR	NR	NR	NR	97.59	NR	NR	[57]
*Gloeocapsa* sp. N107	NR	NR	Salt lake	2.2 M	38	Photoautotrophic	NR	NR	NR	NR	NR	NR	NR	[56]
*Gloeocapsa* sp. CU-2556	NR	NR	Stone monument	NR	23	Photoautotrophic	✓	NR	NR	NR	NR	NR	NR	[68]
*Gloeocapsa* sp. strain C-90-Cal-G	NR	NR	Limestone quarry wall	NR	NR	Photoautotrophic	✓	NR	NR	NR	NR	NR	NR	[67]

a Genotypic data are presented in comparison to *Gloeocapsa* sp. strain BRSZ.

b Biosynthetic gene cluster of mycosporine-like amino acids (BGC of MAAs) was identified using antiSMASH. The core BGC of MAAs comprises *MysA, MysB, and MysC*, encoding dehydroquinate synthase, O-methyltransferase, and an ATP-grasp enzyme, respectively.✓ = MAA detected in the strain.NR = No reported.

### 3.5 Secondary structure of 16S–23S rRNA ITS

**Fig. 4:**
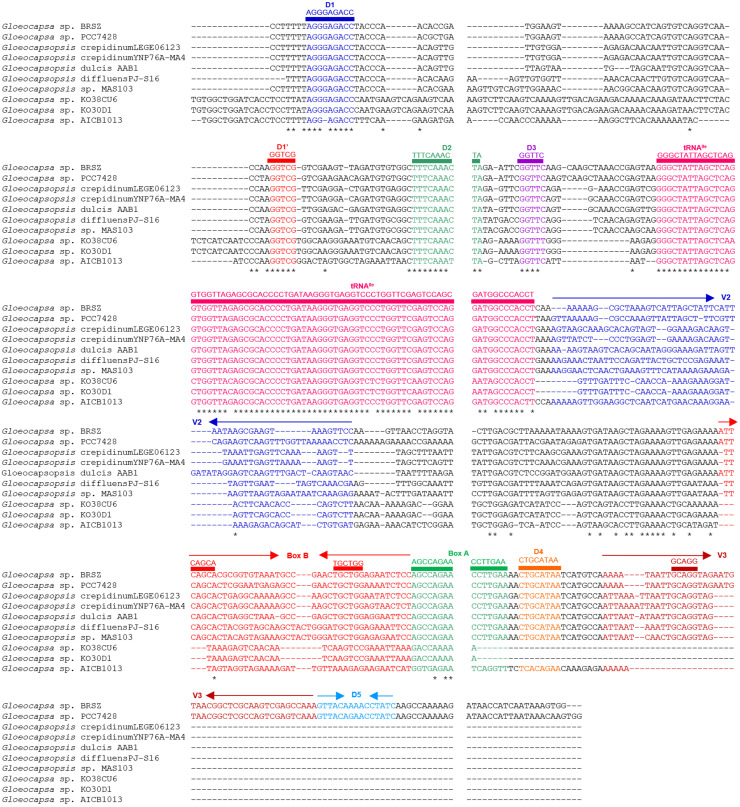
**Alignment of the nucleotide sequences of the 16S–23S ITS regions of strain BRSZ and nine focal strains within the genera *Gloeocapsa* and *Gloeocapsopsis*.** The conserved domains D1, D1′, D2-D5, tRNA^Ile^, Box A, and the major variable regions V2, Box B, and V3 are labeled above the sequences.

**Fig. 5:**
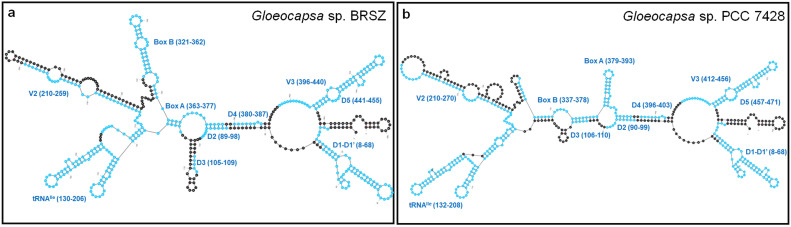
**Secondary structure of the 16S rRNA–23S rRNA ITS region of (a) *Gloeocapsa* sp. strain BRSZ and (b) *Gloeocapsa* sp. PCC 7428.** The identified fragments, D1-D1′, D2, D3, tRNA^Ile^, V2, Box B, Box A, D4, V3, and D5, are marked in blue.

**Fig. 6:**
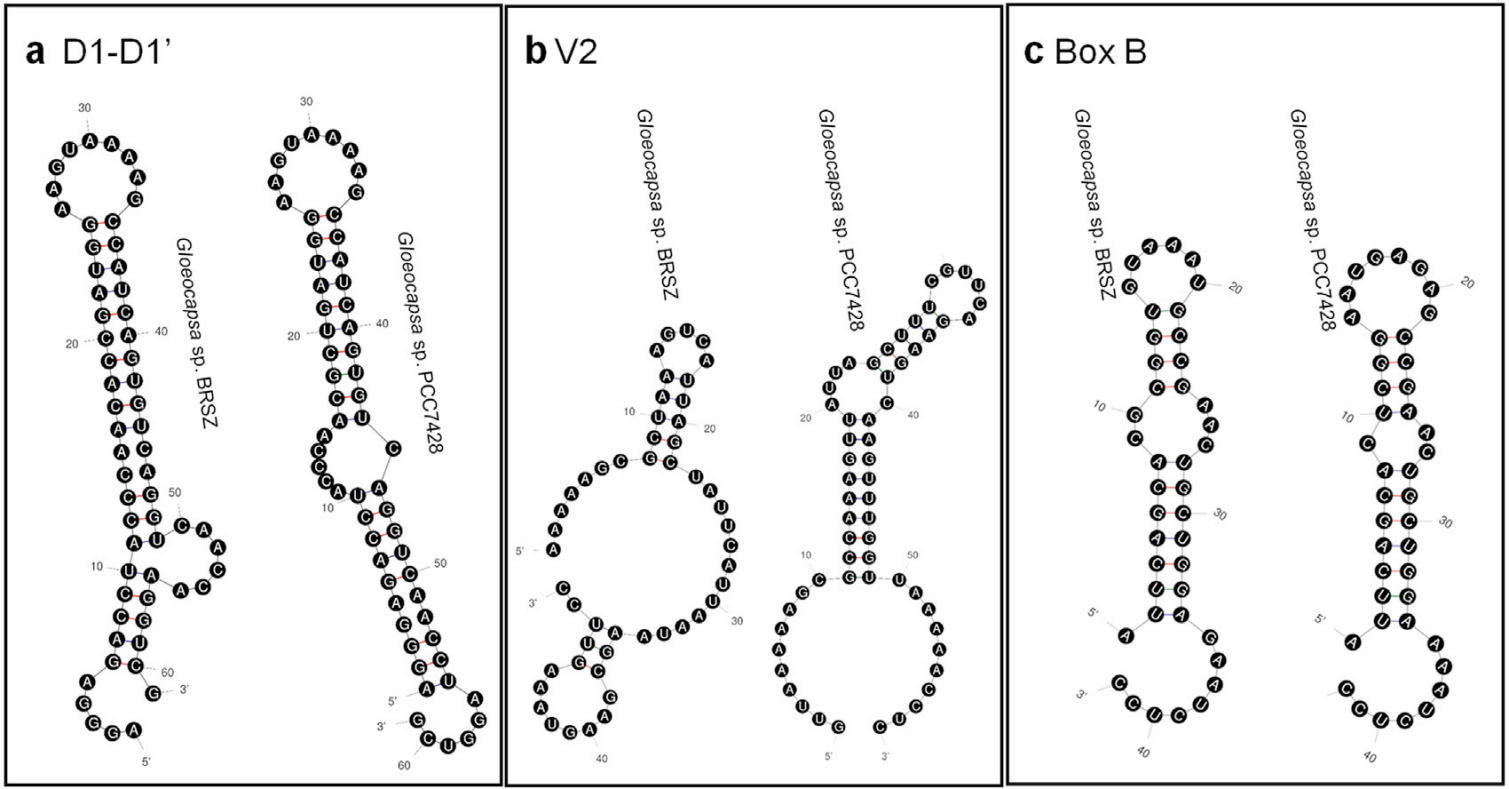
Predicted secondary structures of (a) D1-D1′, (b) V2, and (c) Box B region within the 16S–23S ITS of *Gloeocapsa* sp. BRSZ and *Gloeocapsa* sp. PCC7428.

The 16S–23S rRNA ITS sequences of strain BRSZ and nine representative focal strains within the genera *Gloeocapsa* and *Gloeocapsopsis* were used to predict the secondary structures. The lengths of the 16S–23S ITS sequences ranged from 393 to 504 bp (Supplementary Table S2). These sequences exhibited a consistent organizational pattern, featuring one tRNA^Ile^ and varying lengths of other fragments in the rRNA gene cluster among the ten representative strains (Supplementary Figure S4). Aligning all ITS sequences via CLUSTALW revealed conserved domains, including D1, D1′, D2-D5, tRNA^Ile^, Box A, and major variable regions V2, Box B, and V3 (Fig. 4). The fragments were identified based on the ITS region of *Nostoc* PCC 7120 [39]. The ITS sequence of BRSZ shared 81.03 % similarity with *Gloeocapsa* sp. PCC 7428. Secondary structure predictions showed slight differences, particularly in the D1-D1′, V2, and Box B regions (Fig. 5). Notably, variations in individual fragment lengths, such as D1-D1′ (61–98 bp), V2 (45–64 bp), and Box B (31–44 bp), among the ten representative taxa led to significant differences in secondary structures (Supplementary Table S2). Consequently, the secondary structures of these fragments were individually predicted (Fig. 6 and Supplementary Figure S5).

Despite the similarity between strains BRSZ and *Gloeocapsa* sp. PCC 7428, the D1-D1′ structures of strain BRSZ differed from those of *Gloeocapsa* sp. PCC 7428 (Fig. 6a). The structure of D1-D1′ in strain BRSZ closely resembled that of *Gloeocapsopsis crepidinum* YNP76A-MA4 and *Gloeocapsopsis dulcis* AAB1 (Supplementary Figure S5a), featuring an exterior loop, a short helix fragmented by a right bulge, a long helix, and ending with a hairpin loop. In contrast, the D1-D1′ of *Gloeocapsa* sp. PCC 7428 terminated with the same hairpin loop, but had two longer helices and included a left bulge (Fig. 6a). Furthermore, the V2 and Box B structures of strain BRSZ displayed distinctive features compared to those of the other strains (Supplementary Figure S5b and S5c). Moreover, the V2 structure of BRSZ differed from that of the other analyzed strains (Supplementary Figure S5b). It consisted of an asymmetrical exterior loop connected to two helices and two hairpin loops. In contrast, the V2 structure of *Gloeocapsa* sp. PCC 7428 has a symmetrical exterior loop with a helix, an interior loop, a short helix, and a hairpin loop (Fig. 6b). Box B of strain BRSZ mainly consists of an exterior helix associated with a long fragment, an asymmetrical loop, a short fragment, and a hairpin loop (Fig. 6c); that of *Gloeocapsa* sp. PCC 7428 had different numbers of residues in the helices, loops, and hairpin loops (Fig. 6c). Considering all conserved domains of ITS, excluding D1-D1′ (D2-D3, tRNA^Ile^, Box A, and V3-D5; Supplementary Figure S5d-S5g), strain BRSZ was the most similar to *Gloeocapsa* sp. PCC 7428 among all the representative strains.

Apart from the differences in the ITS secondary structure between the two strains, their pairwise distance (*p*-distance) was also significantly different (Supplementary Table S3). The percent dissimilarity among the aligned 16S–23S ITS regions of representative strains was calculated as a 100 × *p*-distance. BRSZ showed 11.9 % dissimilarity with *Gloeocapsa* sp. PCC 7428 (Table 1a), with a range of 20.4 to 63.7 % compared with the other strains (Supplementary Table S3). Notably, the percentage dissimilarity can be used to distinguish between populations within the same species (average of ∼1.0 % or less, with all pairwise comparisons showing <3 % dissimilarity) and those representing different species (>7 % dissimilarity). However, when the difference ranges between 3 and 7 %, the cutoff is unclear; therefore, decisions should be based on other criteria [63]. Because strain BRSZ showed >7 % dissimilarity from the other strains, it can be classified as a different species.

The secondary structure of the 16S–23S ITS in cyanobacteria serves as a critical tool for species-level identification, aiding in the discovery of new species and differentiation of taxa [33,56,64]. However, there is a lack of information regarding the secondary structures of *Gloeocapsa* spp. in public databases. Recently, the specificity and sensitivity of 16S–23S ITS sequences have been pivotal in classifying novel species in *Gloeocapsopsis* [33], a sister taxon closely related to strain BRSZ. The comparison of secondary structures of the D1-D1′ and Box B regions of *Gloeocapsopsis* spp. revealed slight structural differences. The *p*-distance indicated a dissimilarity greater >15 % among *Gloeocapsopsis* spp., exceeding the proposed 7 % cutoff for species differentiation [63]. These findings underscore the effectiveness of utilizing the 16S–23S ITS secondary structure for identifying novel species. Detailed analysis of the 16S–23S ITS secondary structures proved effective in species-level identification, leading to the classification of strain BRSZ as a new species within the genus *Gloeocapsa*.

### 3.6 UV-absorbing compounds

**Fig. 7:**
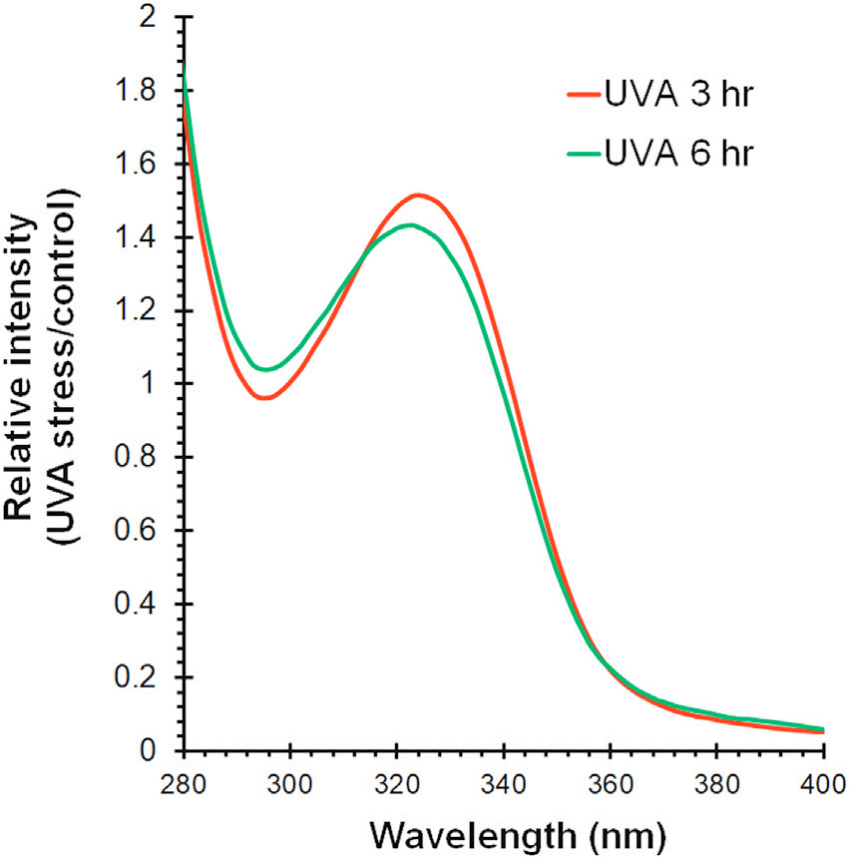
**UV–Vis spectra of UV-absorbing compounds in the aqueous phase extracted from *Gloeocapsa* sp. strain BRSZ**. The strain BRSZ was exposed to UV light for 0 (control), 3, or 6 h. The spectra shown display the relative absorbance intensity normalized to the control across the UV–Vis range.

Secondary metabolites produced by cyanobacteria, such as mycosporine-like amino acids (MAAs), play crucial roles in environmental adaptation. These metabolites have been found to possess photoprotective properties against UV radiation and act as biological sunscreens [65]. Whole-genome analysis of strain BRSZ revealed the presence of an MAA biosynthetic gene cluster, which includes a 2-epi-5-epi-valiolone synthase, an *O*-methyltransferase, and an ATP-grasp enzyme (GenBank: JBEGHC000000000.1) [40]. To determine whether the strain accumulated UV-absorbing compounds, BRSZ cells were extracted after 3 and 6 h of UV exposure, and the UV–Vis absorption spectra were obtained. The aqueous solution obtained, containing partially purified UV-absorbing compounds, revealed an absorption peak at approximately 325–330 nm at both exposure times (Fig. 7), indicating that BRSZ adapts to UV stress by producing UV-absorbing compounds.

Several organisms, including cyanobacteria, exposed to UV radiation in their habitats have evolved to accumulate UV-absorbing compounds, such as MAAs, to counteract the negative effects of UV radiation. Accumulation of MAAs has been reported in a wide range of cyanobacterial species [66,67], with reports of MAA biosynthesis by the genus *Gloeocapsa* [68,69]. For instance, the *Gloeocapsa* sp. strain C-90-Cal-G accumulates MAAs with an absorbance maximum at 326 nm, which is directly related to the UV radiation intensity [68]. Additionally, UV-B radiation significantly influences MAA biosynthesis in *Gloeocapsa* sp. CU-2556 in a dose-dependent manner, leading to the accumulation of shinorine and M-307, with absorption maxima at 333 and 307 nm, respectively [69]. To date, there have been no reports on MAA sources in *Gloeocapsopsis* [70]. These findings highlight the adaptive response of the genus *Gloeocapsa* spp. to UV radiation via MAA production as a protective mechanism against UV-induced damage. In modern cyanobacterial classification systems, the polyphasic approach emphasizes the importance of secondary metabolites along with genetic, morphological, and ecological criteria [71]. Given the morphological similarities between cyanobacteria in the *Gloeocapsa* and *Gloeocapsopsis* genera, our findings on MAA production by strain BRSZ, along with existing literature on *Gloeocapsa* spp., provide evidence supporting the classification of strain BRSZ as a new species within the genus *Gloeocapsa*.

## 4 Conclusion

In conclusion, this polyphasic approach, which integrates morphological, physiological, genetic, and ecological data, robustly supports the classification of BRSZ as a new species in the genus *Gloeocapsa*. Morphological characterization revealed distinctive features consistent with *Gloeocapsa*, whereas physiological studies demonstrated its extremophilic nature, showing its ability to withstand high temperatures, salinity, and UV radiation. Phylogenetic analysis based on the 16S rRNA gene placed BRSZ within a well-defined clade alongside *Gloeocapsa* species, a finding corroborated by ITS secondary structure analysis that differentiated it from closely related genera, such as *Gloeocapsopsis*. Furthermore, the production of UV-absorbing compounds under UV exposure highlighted its environmental adaptation strategy, which is similar to that of other *Gloeocapsa* species. Overall, this approach led to the identification of BRSZ as *Gloeocapsa* sp. BRSZ. Moreover, these findings underscore the importance of a polyphasic approach for accurately characterizing and classifying novel cyanobacterial species and provide insights into their ecological roles and potential applications in biotechnology. Future genomic and metabolic studies will further elucidate the unique adaptations and contributions of the BRSZ to microbial diversity.

## Data Availability Statement

The data supporting the findings of this study are included in this published article as well as in the Supplementary Materials available online.

## CRediT authorship contribution statement

**Sasiprapa Samsri:** Writing – original draft, Formal analysis. **Tanwalee Deprom:** Investigation. **Chananwat Kortheerakul:** Investigation. **Sophon Sirisattha:** Investigation. **Stephen B. Pointing:** Writing – review & editing, Funding acquisition, Conceptualization. **Hakuto Kageyama:** Writing – review & editing, Funding acquisition, Conceptualization. **Rungaroon Waditee-Sirisattha:** Writing – review & editing, Funding acquisition, Conceptualization.

## Declaration of Competing Interes

The authors declare that they have no known competing financial interests or personal relationships that could have appeared to influence the work reported in this paper.

## Appendix Supplementary materials

Image, application 1

Image, application 2

## References

[1] Malavasi V., Soru S., Cao G. (2020). Extremophile microalgae: the potential for biotechnological application. J. Phycol..

[2] Demoulin C.F., Lara Y.J., Cornet L., Francois C., Baurain D., Wilmotte A., Javaux E.J. (2019). Cyanobacteria evolution: insight from the fossil record. Free Radic. Biol. Med..

[3] Bolhuis H., Cretoiu M.S., Stal L.J. (2014). Molecular ecology of microbial mats. FEMS Microbiol. Ecol..

[4] Hagemann M. (2011). Molecular biology of cyanobacterial salt acclimation. FEMS Microbiol. Rev..

[5] Thomas D.N. (2005). Photosynthetic microbes in freezing deserts. Trends. Microbiol..

[6] Kageyama H., Waditee-Sirisattha R. (2023). Halotolerance mechanisms in salt‑tolerant cyanobacteria. Adv. Appl. Microbiol..

[7] Van den Burg B. (2003). Extremophiles as a source for novel enzymes. Curr. Opin. Microbiol..

[8] Whitton B.A. (2012).

[9] Pedersen D., Miller S.R. (2017). Photosynthetic temperature adaptation during niche diversification of the thermophilic cyanobacterium Synechococcus A/B clade. ISME.

[10] Purcell D., Sompong U., Yim L.C., Barraclough T.G., Peerapornpisal Y., Pointing S.B. (2007). The effects of temperature, pH and sulphide on the community structure of hyperthermophilic streamers in hot springs of northern Thailand. FEMS Microbiol. Ecol..

[11] Fecteau K.M., Boyd E.S., Lindsay M.R., Amenabar M.J., Robinson K.J., Debes R.V., Shock E.L. (2021). Cyanobacteria and algae meet at the limits of their habitat ranges in moderately acidic hot springs. J. Geophys. Res.-Biogeosci..

[12] Klanchui A., Cheevadhanarak S., Prommeenate P., Meechai A. (2017). Exploring components of the CO(2)-concentrating mechanism in alkaliphilic cyanobacteria through genome-based analysis. Comput. Struct. Biotechnol. J..

[13] Grant W.J.T.W.D., Herbert T.A., Codd G.A. (1986).

[14] Amarouche-Yala S., Benouadah A., El Ouahab Bentabet A., Lopez-Garcia P. (2014). Morphological and phylogenetic diversity of thermophilic cyanobacteria in Algerian hot springs. Extremophiles.

[15] Keshari N., Zhao Y., Das S.K., Zhu T., Lu X. (2022). Cyanobacterial community structure and isolates from representative hot springs of Yunnan Province, China using an integrative approach. Front. Microbiol..

[16] Yilmaz CankiliÇ M. (2016). Determination of cyanobacterial composition of eynal (Sİmav) Hot Spring in KÜtahya, Turkey. Appl. Ecol. Environ. Res..

[17] Strunecky O., Kopejtka K., Goecke F., Tomasch J., Lukavsky J., Neori A., Kahl S., Pieper D.H., Pilarski P., Kaftan D., Koblizek M. (2019). High diversity of thermophilic cyanobacteria in Rupite hot spring identified by microscopy, cultivation, single-cell PCR and amplicon sequencing. Extremophiles.

[18] Ikram S.F., Uniyal V., Kumar D. (2021). Changes in species composition of cyanobacterial and microalgal communities along a temperature gradient in Tapovan Hot Spring, Garhwal Himalaya, Uttarakhand, India. Aquat. Ecol..

[19] Martinez J.N., Nishihara A., Lichtenberg M., Trampe E., Kawai S., Tank M., Kuhl M., Hanada S., Thiel V. (2019). Vertical distribution and diversity of phototrophic bacteria within a hot spring microbial mat (Nakabusa Hot Springs, Japan). Microbes Environ..

[20] McGregor G.B., Rasmussen J.P. (2008). Cyanobacterial composition of microbial mats from an Australian thermal spring: a polyphasic evaluation. FEMS Microbiol. Ecol..

[21] Sharp C.E., Brady A.L., Sharp G.H., Grasby S.E., Stott M.B., Dunfield P.F. (2014). Humboldt’s spa: microbial diversity is controlled by temperature in geothermal environments. ISME.

[22] Boomer S.M., Noll K.L., Geesey G.G., Dutton B.E. (2009). Formation of multilayered photosynthetic biofilms in an alkaline thermal spring in Yellowstone National Park, Wyoming. Appl. Environ. Microbiol..

[23] Bennett A.C., Murugapiran S.K., Hamilton T.L. (2020). Temperature impacts community structure and function of phototrophic Chloroflexi and Cyanobacteria in two alkaline hot springs in Yellowstone National Park. Environ. Microbiol. Rep..

[24] Brenes-Guillen L., Vidaurre-Barahona D., Morales S., Mora-Lopez M., Sittenfeld A., Uribe-Lorio L. (2021). Novel cyanobacterial diversity found in Costa Rican thermal springs associated with Rincon de la Vieja and Miravalles volcanoes: a polyphasic approach. J. Phycol..

[25] Lau M.C., Aitchison J.C., Pointing S.B. (2009). Bacterial community composition in thermophilic microbial mats from five hot springs in central Tibet. Extremophiles.

[26] Subtavewung P.-h., Raksaskulwong M., Tulyatid J. (2005). Proceedings World Geothermal Congress.

[27] Hongmei J., Aitchison J.C., Lacap D.C., Peerapornpisal Y., Sompong U., Pointing S.B. (2005). Community phylogenetic analysis of moderately thermophilic cyanobacterial mats from China, the Philippines and Thailand. Extremophiles.

[28] Sompong U., Hawkins P.R., Besley C., Peerapornpisal Y. (2005). The distribution of cyanobacteria across physical and chemical gradients in hot springs in northern Thailand. FEMS Microbiol. Ecol..

[29] Sompong U., Castenholz R.W., Anuntalabhochai S., Peerapornpisal Y. (2006). Genetical diversity of mastigocladus in Ranong hot spring, Southern Part of Thailand. Chiang Mai J. Sci..

[30] Rippka R., Deruelles J., Waterbury J.B., Herdman M., Stanier R.Y. (1979). Generic assignments, strain histories, and properties of pure cultures of Cyanobacteria. Microbiol.

[31] Debnath M., Mandal N.C., Ray S. (2009). The study of cyanobacterial flora from geothermal springs of Bakreswar, West Bengal, India. Algae.

[32] Adar O., Kaplan-Levy R.N., Schonhölz M., Ronen B., Banet G. (2017). Optimal temperature and salinity for growth of two Cyanobacteria from Hammat Gader hot springs. Negev, Dead Sea Arava Stud..

[33] Jung P., Azua-Bustos A., Gonzalez-Silva C., Mikhailyuk T., Zabicki D., Holzinger A., Lakatos M., Budel B. (2021). Emendation of the coccoid Cyanobacterial Genus Gloeocapsopsis and Description of the New Species Gloeocapsopsis diffluens sp. nov. and Gloeocapsopsis dulcis sp. nov. Isolated From the Coastal Range of the Atacama Desert (Chile). Front. Microbiol..

[34] Casamatta D.A., Villanueva C.D., Garvey A.D., Stocks H.S., Vaccarino M., Dvorak P., Hasler P., Johansen J.R. (2020). Reptodigitus Chapmanii (Nostocales, Hapalosiphonaceae) Gen. Nov.: a unique nostocalean (Cyanobacteria) genus based on a polyphasic approach(1). J. Phycol..

[35] Konstantinou D., Voultsiadou E., Panteris E., Gkelis S. (2021). Revealing new sponge-associated cyanobacterial diversity: novel genera and species. Mol. Phylogenet. Evol..

[36] Strunecky O., Raabova L., Bernardova A., Ivanova A.P., Semanova A., Crossley J., Kaftan D. (2020). Diversity of cyanobacteria at the Alaska North Slope with description of two new genera: Gibliniella and Shackletoniella. FEMS Microbiolol. Ecol..

[37] George C., Kortheerakul C., Khunthong N., Sharma C., Luo D., Chan K.G., Daroch M., Hyde K.D., Lee P.K.H., Goh K.M., Waditee-Sirisattha R., Pointing S.B. (2025). Spatial scale modulates stochastic and deterministic influence on biogeography of photosynthetic biofilms in Southeast Asian hot springs. Environ. Microbiome.

[38] O'Toole G.A. (2016). Classic spotlight: how the gram stain works. J. Bacteriol. Res..

[39] Iteman I., Rippka R., Tandeau de Marsac N., Herdman M. (2000). Comparison of conserved structural and regulatory domains within divergent 16S rRNA–23S rRNA spacer sequences of cyanobacteria. Microbiol.

[40] Aono T., Samsri S., Waditee-Sirisattha R., Kageyama H. (2024). Draft genome sequence of a cyanobacterium Gloeocapsa sp. BRSZ, isolated from Bo Khlueng hot spring in Ratchaburi, Thailand. Microbiol. Resour. Announc..

[41] Tamura K., Stecher G., Kumar S. (2021). MEGA11: molecular evolutionary genetics analysis version 11. Mol. Biol. Evol..

[42] Saitou N., Nei M. (1987). The neighbor-joining method: a new method for reconstructing phylogenetic trees. Mol. Biol. Evol..

[43] Felsenstein J. (1985). Confidence limits on phylogenies: an approach using the bootstrap. Evolution.

[44] Tamura K., Nei M., Kumar S. (2004). Prospects for inferring very large phylogenies by using the neighbor-joining method. Proc. Natl. Acad. Sci..

[45] Richter M., Rossello-Mora R., Oliver Glockner F., Peplies J. (2016). JSpeciesWS: a web server for prokaryotic species circumscription based on pairwise genome comparison. Bioinformatics.

[46] Richter M., Rosselló-Móra R. (2009). Shifting the genomic gold standard for the prokaryotic species definition. Proc. Natl. Acad. Sci..

[47] Meier-Kolthoff J.P., Carbasse J.S., Peinado-Olarte R.L., Goker M. (2022). TYGS and LPSN: a database tandem for fast and reliable genome-based classification and nomenclature of prokaryotes. Nucleic Acids Res..

[48] Jan P Meier-Kolthoff A.F.A., Klenk Hans-Peter, Göker Markus (2013). Genome sequence-based species delimitation with confidence intervals and improved distance functions. BMC Bioinformatics.

[49] Blin K., Shaw S., Augustijn H.E., Reitz Z.L., Biermann F., Alanjary M., Fetter A., Terlouw B.R., Metcalf W.W., Helfrich E.J.N., van Wezel G.P., Medema M.H., Weber T. (2023). antiSMASH 7.0: new and improved predictions for detection, regulation, chemical structures and visualisation. Nucleic Acids Res..

[50] Zuker M. (2003). Mfold web server for nucleic acid folding and hybridization prediction. Nucleic Acids Res..

[51] Ngoennet S., Nishikawa Y., Hibino T., Waditee-Sirisattha R., Kageyama H. (2018). A method for the isolation and characterization of mycosporine-like amino acids from cyanobacteria. Methods Protoc..

[52] Herdman M., Castenholz R.W., Iteman I., Waterbury J.B., Rippka R., Boone D.R., Castenholz R.W., Garrity G.W. (2001). Bergey’s Manual of Systematic bacteriology. 2:1. The archaea, Cyanobacteria and Deeply Branched Bacteria.

[53] Stebegg R., Schmetterer G., Rompel A. (2023). Heterotrophy among Cyanobacteria. ACS Omega.

[54] Mataloni G., Komárek J. (2004). Gloeocapsopsis aurea, a new subaerophytic cyanobacterium from maritime Antarctica. Polar Biol..

[55] Azua-Bustos A., Zuniga J., Arenas-Fajardo C., Orellana M., Salas L., Rafael V. (2014). Gloeocapsopsis AAB1, an extremely desiccation-tolerant cyanobacterium isolated from the Atacama Desert. Extremophiles.

[56] Kaštovský J., Gomez E.B., Hladil J., Johansen J.R. (2014). Cyanocohniella calida gen. et sp. nov. (Cyanobacteria: aphanizomenonaceae) a new cyanobacterium from the thermal springs from Karlovy Vary, Czech Republic. Phytotaxa.

[57] Mackay M.A., Norton R.S., Borowitzka L.J. (1984). Organic osmoregulatory solutes in cyanobacteria. Microbiol.

[58] Casero M.C., Herrero M.A., De la Roche J.P., Quesada A., Velazquez D., Cires S. (2024). Effect of salinity on scytonemin yield in endolithic cyanobacteria from the Atacama Desert. Sci. Rep..

[59] Komárek J., Kaštovský J., Mareš J., Johansen J.R. (2014). Taxonomic classification of cyanoprokaryotes (cyanobacterial genera) 2014, using a polyphasic approach. Preslia.

[60] Komárek J. (2016). A polyphasic approach for the taxonomy of cyanobacteria: principles and applications. Eur. J. Phycol..

[61] Stackebrandt E.a.E., E J. (2006). Taxonomic parameters revisited: tarnished gold standards. Microbiol. Today.

[62] Auch A.F., Klenk H.P., Goker M. (2010). Standard operating procedure for calculating genome-to-genome distances based on high-scoring segment pairs. Stand Genomic Sci..

[63] Gonzalez-Resendiz L., Johansen J.R., Leon-Tejera H., Sanchez L., Segal-Kischinevzky C., Escobar-Sanchez V., Morales M. (2019). A bridge too far in naming species: a total evidence approach does not support recognition of four species in Desertifilum (Cyanobacteria). J. Phycol..

[64] McGovern C.A., Norwich A.R., Thomas A.L., Hamsher S.E., Biddanda B.A., Weinke A.D., Casamatta D.A. (2023). Unbiased analyses of ITS folding motifs in a taxonomically confusing lineage: anagnostidinema visiae sp. nov. (cyanobacteria). J. Phycol..

[65] Kageyama H., Waditee-Sirisattha R. (2018).

[66] Garcia-Pichel F., Castenholz R.W. (1993). Occurrence of UV-absorbing, mycosporine-like compounds among cyanobacterial isolates and an estimate of their screening capacity. Appl. Environ. Microbiol..

[67] Jain S., Prajapat G., Abrar M., Ledwani L., Singh A., Agrawal A. (2017). Cyanobacteria as efficient producers of mycosporine-like amino acids. J. Basic Microbiol..

[68] Garcia-Pichel F., Wingard C.E., Castenholz R.W. (1993). Evidence regarding the UV sunscreen role of a mycosporine-like compound in the Cyanobacterium *Gloeocapsa*sp. Appl. Environ. Microbiol..

[69] Rastogi R.P., Incharoensakdi A. (2014). UV radiation-induced biosynthesis, stability and antioxidant activity of mycosporine-like amino acids (MAAs) in a unicellular cyanobacterium Gloeocapsa sp. CU2556. J. Photochem. Photobiol. B.

[70] Peng J., Guo F., Liu S., Fang H., Xu Z., Wang T. (2023). Recent advances and future prospects of mycosporine-like amino acids. Molecules.

[71] Komárek J. (2017). Several problems of the polyphasic approach in the modern cyanobacterial system. Hydrobiologia.

