# Supplementary material for: Genotypic and molecular characterization of a moderately thermophilic cyanobacterium, Gloeocapsa sp. strain BRSZ
Source: Eng Microbiol. 2025 Aug 5;5(3):100226. doi: 10.1016/j.engmic.2025.100226 (PMC12967839; doi:10.1016/j.engmic.2025.100226)
Supplement: Supplementary file 1 [file mmc1.pdf]

Supplementary data

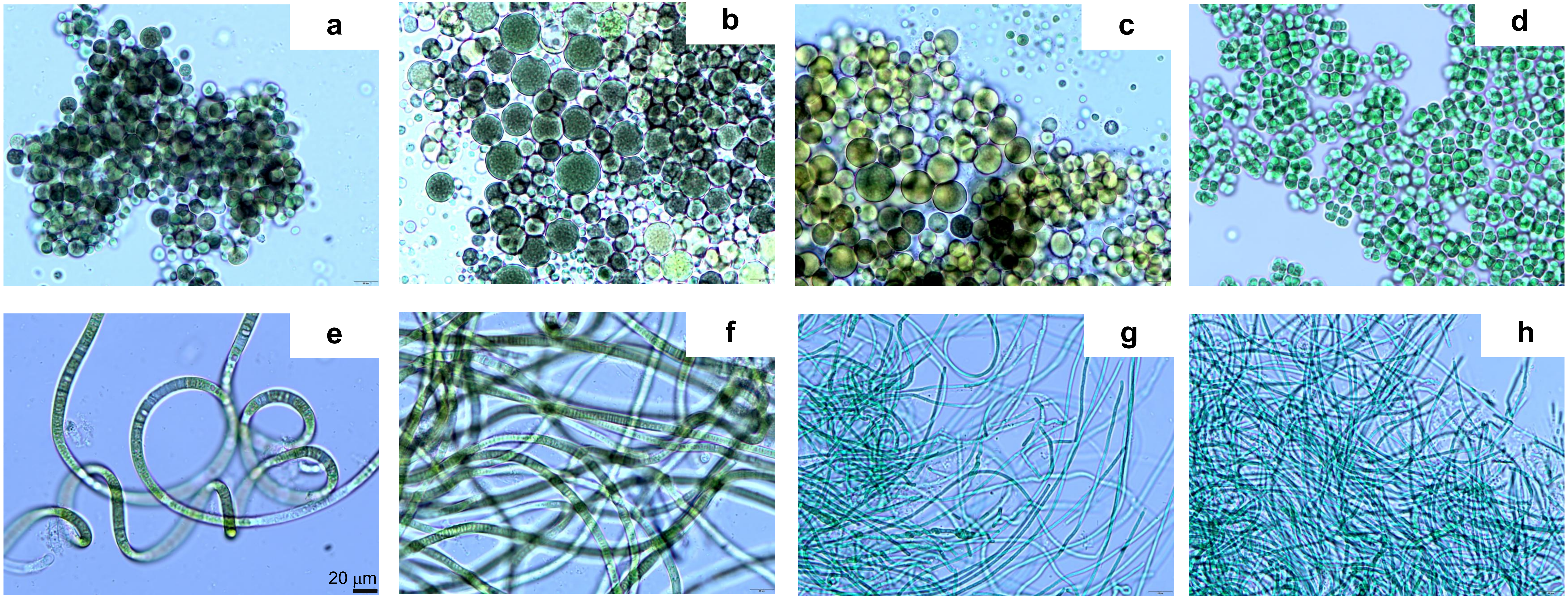

Figure S1 The variation of cyanobacteria isolated from Bo Khlueng hot spring in Thailand at the temperature location number 1 (55 °C). (a-c) Unicellular, (d) colonial, and (e-h) filamentous cyanobacteria.

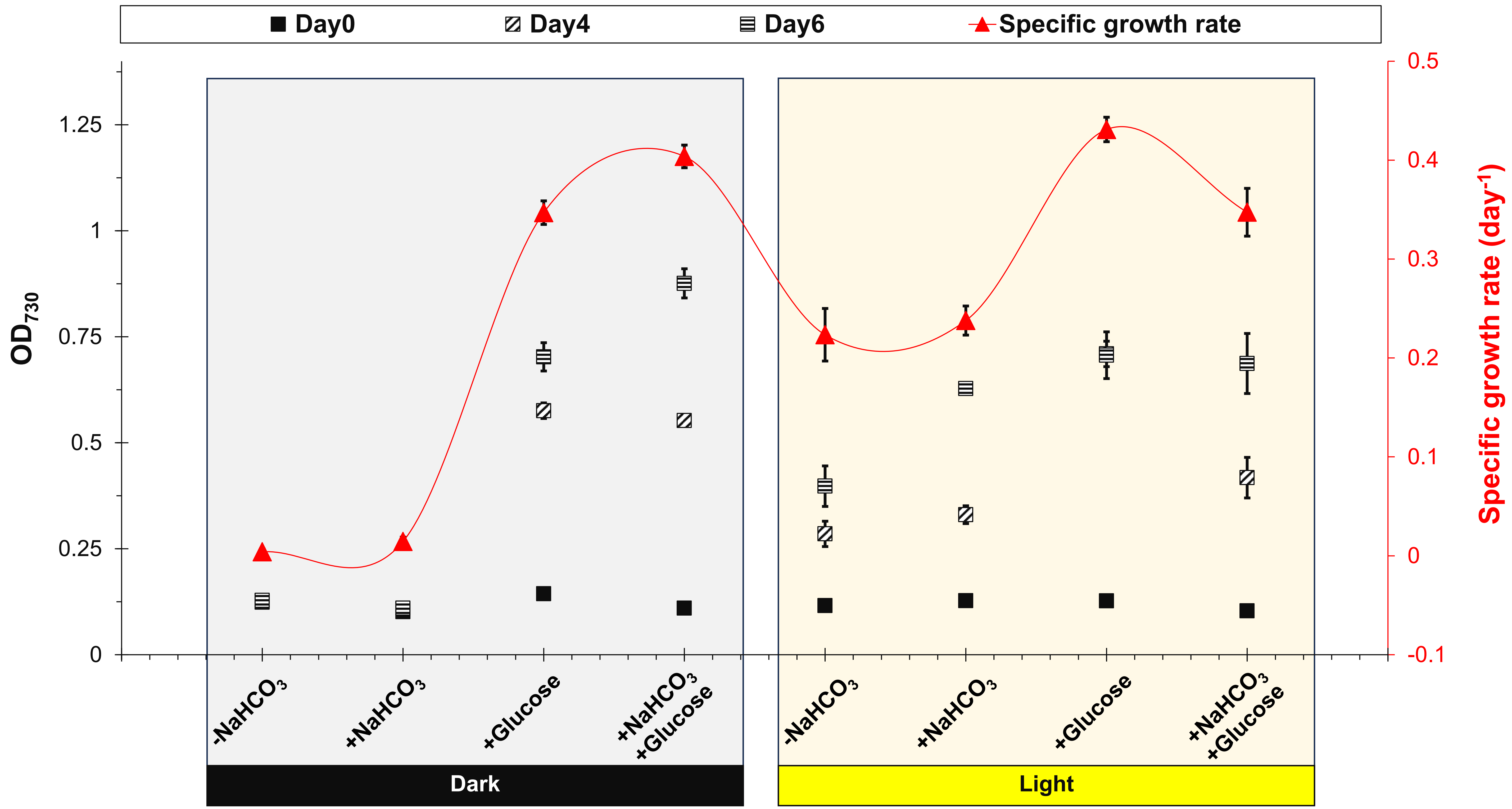

Figure S2 Specific growth rates (day^{-1}) of the strain BRSZ under various nutritional modes. Cultures were grown either in the dark or under continuous light. Carbon sources included sodium bicarbonate (0.4 g L^{-1} of $NaHCO_3$) as an inorganic carbon source and/or glucose (15 g L^{-1}) as an organic carbon source.

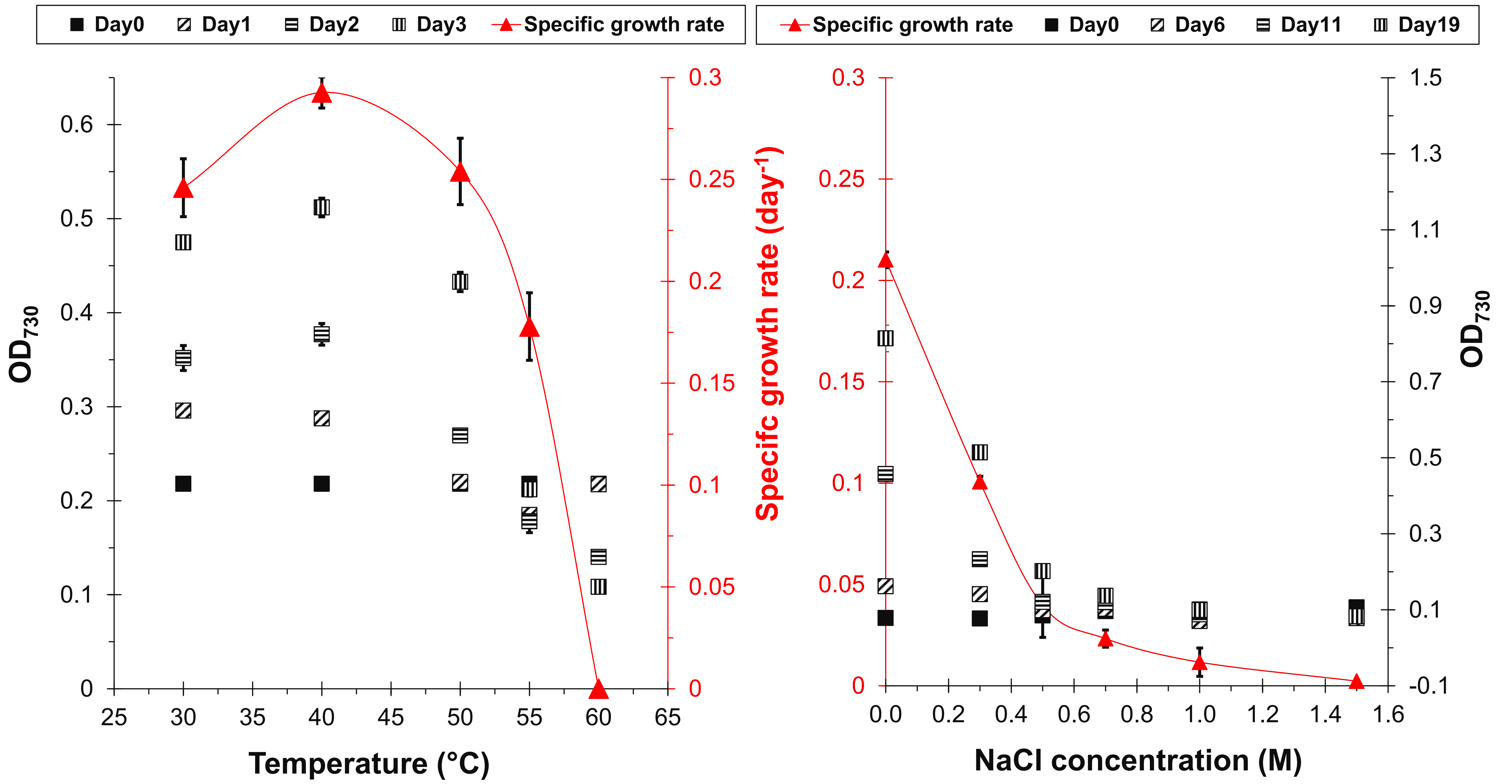

Figure S3 Specific growth rates (day^{-1}) of the strain BRSZ influenced by (Left) temperature (30-60 °C) and (Right) NaCl concentration (0-1.5 M). The specific growth rates were calculated from the change in OD_{730} during the exponential growth phase.

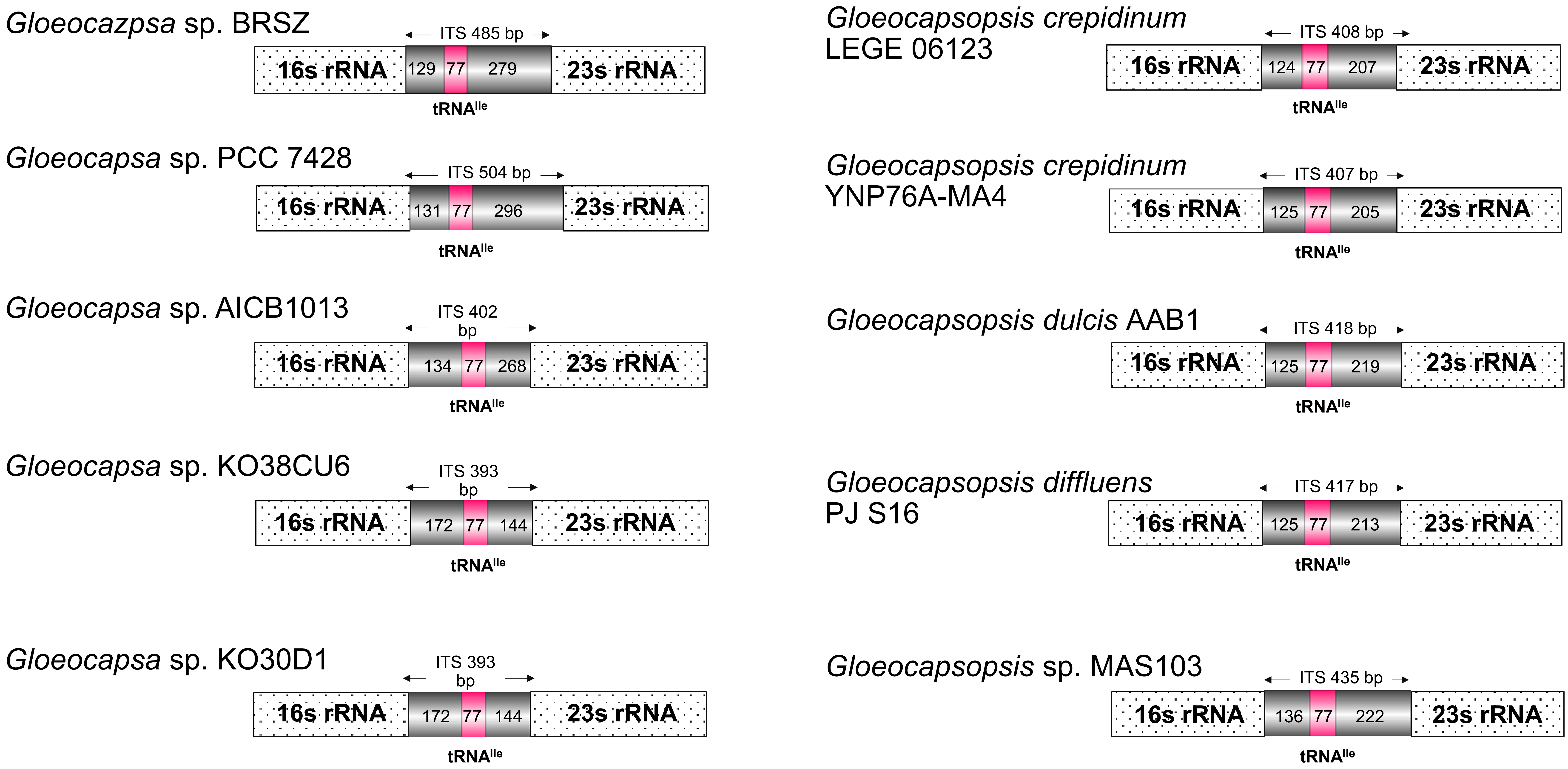

Figure S4 rRNA Gene organization showing ITS region of 10 representative strains. The ITS organization are shown as box in grey color containing $tRNA^{Ile}$ as pink color. The nucleotide length (bp) in each fragment is also shown as the number in the box.

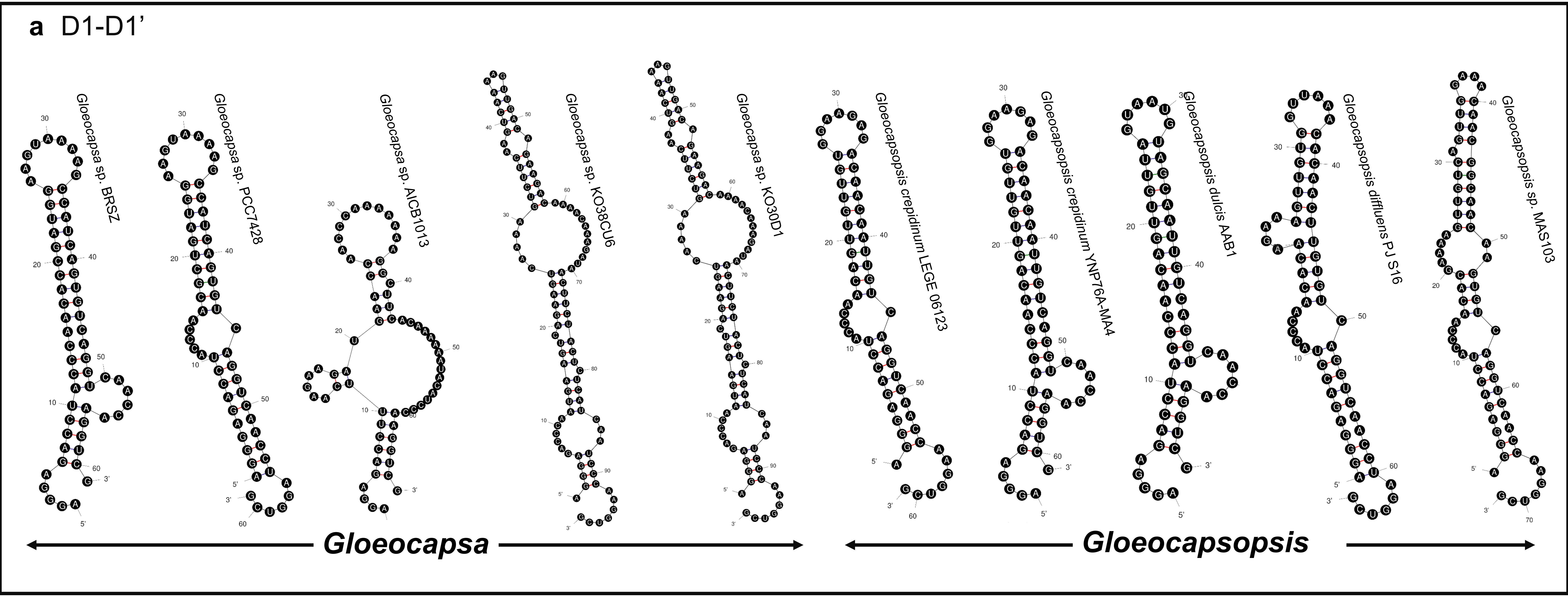

Figure S5 Predicted secondary structures of (a) D1-D1’ (b) V2, (c) Box B, (d) D2-D3, (e) tRNA^{Ile}, (f) Box A, and (g) V3-D5 region within 16S-23S ITS of the strain in genus *Gloeocapsa* and *Gloeocapsopsis*

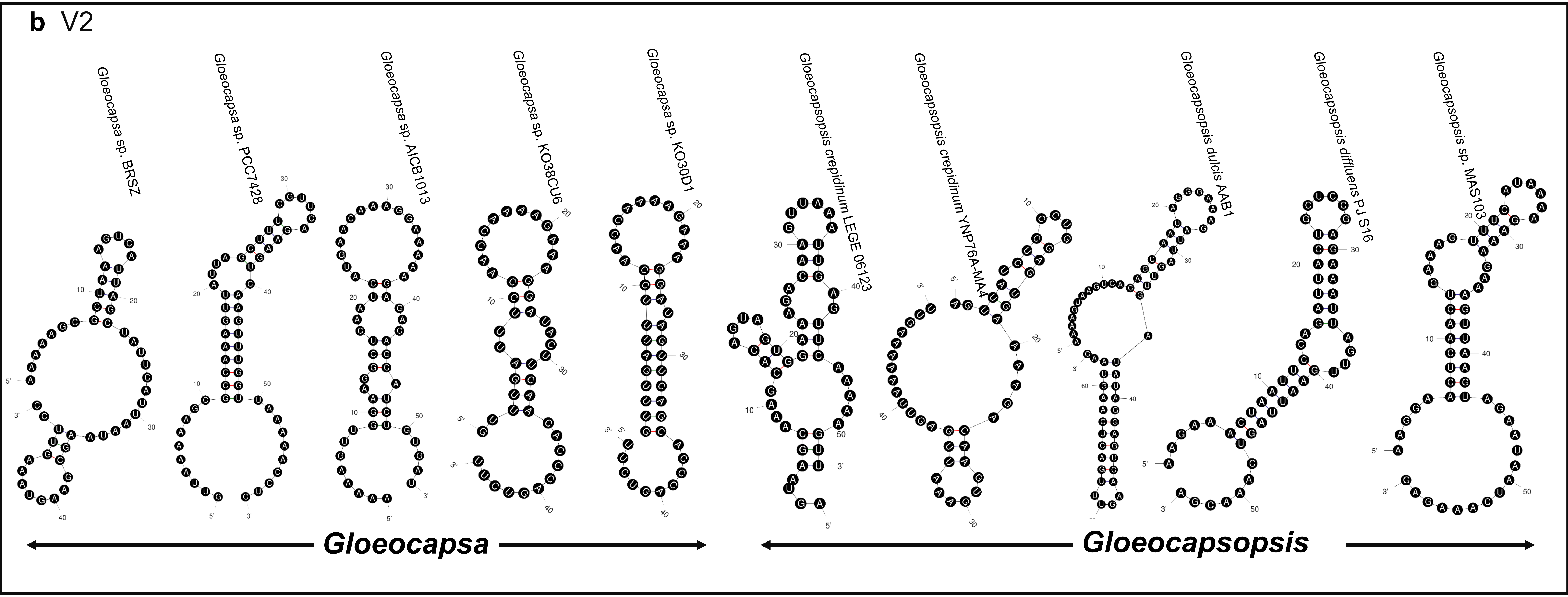
b V2
Gloeocapsa sp. BRSZ
Gloeocapsa sp. PCC7428
Gloeocapsa sp. AICB1013
Gloeocapsa sp. KO38CU6
Gloeocapsa sp. KO30D1
Gloeocapsopsis crepidinum LEGE 06123
Gloeocapsopsis crepidinum YNP76A-MA4
Gloeocapsopsis dulcis AAB1
Gloeocapsopsis diffluens PJ S16
Gloeocapsopsis sp. MAS103
Gloeocapsa
Gloeocapsopsis

c Box B

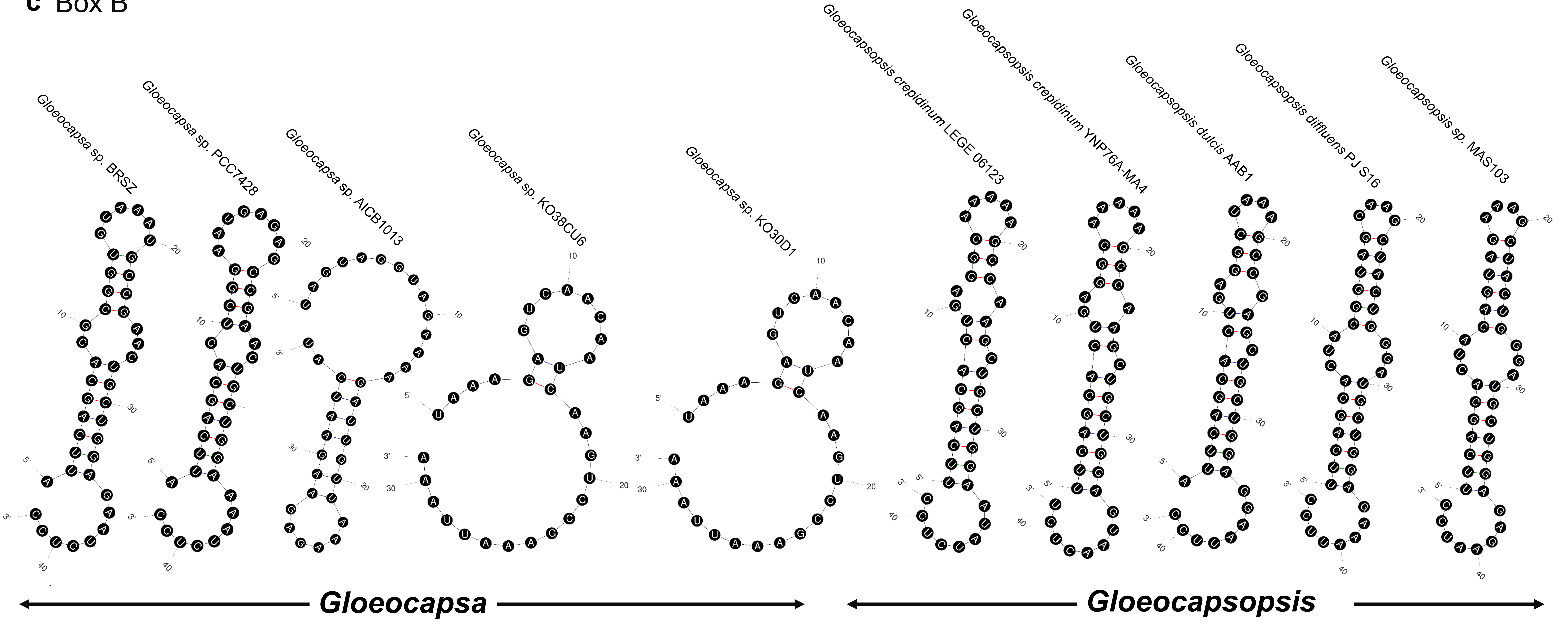
Gloeocapsa sp. BRSZ
Gloeocapsa sp. PCC7428
Gloeocapsa sp. AICB1013
Gloeocapsa sp. KO38CU6
Gloeocapsa sp. KO30D1
Gloeocapsopsis crepidinum LEGE 06123
Gloeocapsopsis crepidinum YNP76A-MA4
Gloeocapsopsis dulcis AAB1
Gloeocapsopsis diffluens PJ S16
Gloeocapsopsis sp. MAS103
Gloeocapsa
Gloeocapsopsis

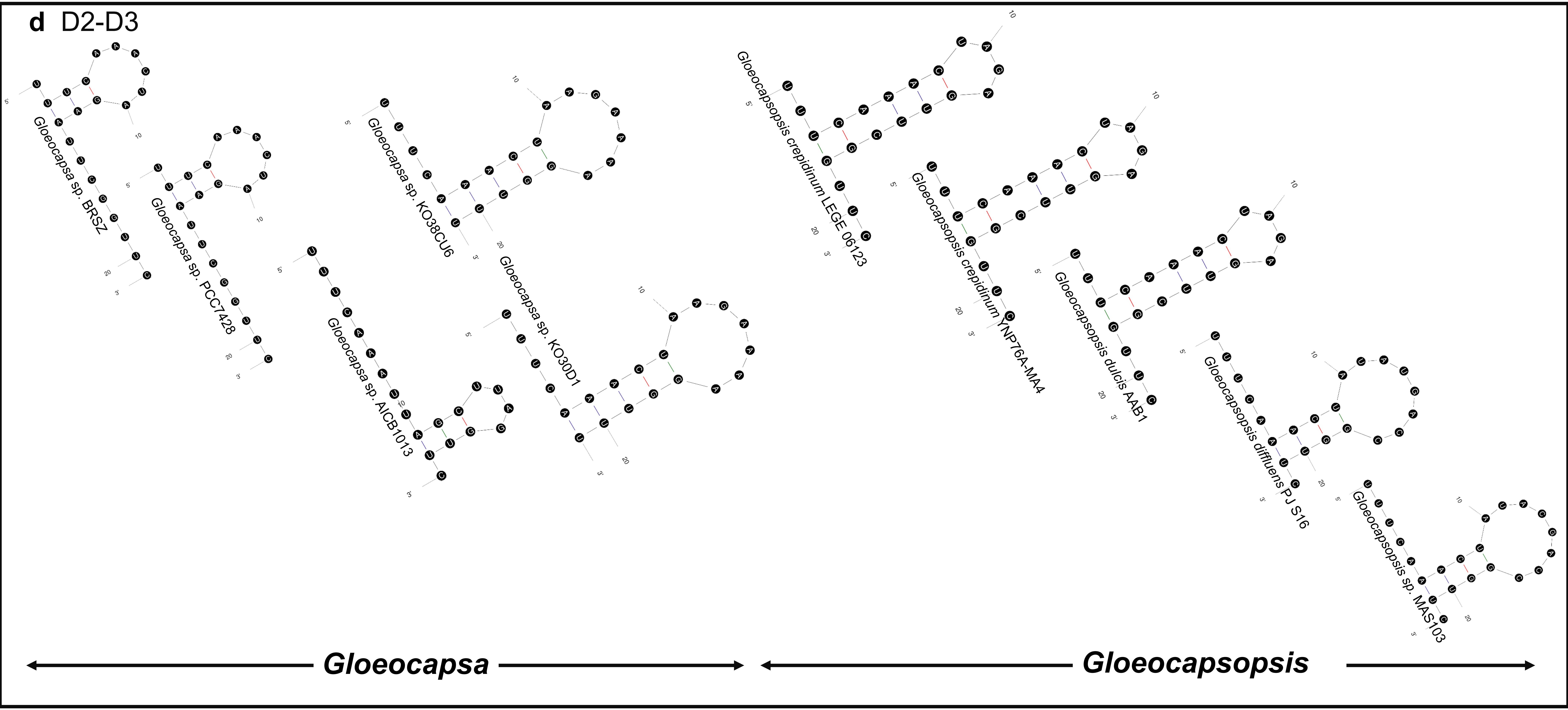
d D2-D3
Gloeocapsa sp. BRSZ
Gloeocapsa sp. PCC7428
Gloeocapsa sp. KO38CU6
Gloeocapsa sp. AICB1013
Gloeocapsa sp. KO30D1
Gloeocapsopsis crepidinum LEGE 06123
Gloeocapsopsis crepidinum YNP76A-MA4
Gloeocapsopsis dulcis AAB1
Gloeocapsopsis diffluens PJ S16
Gloeocapsopsis sp. MAS103
Gloeocapsa
Gloeocapsopsis

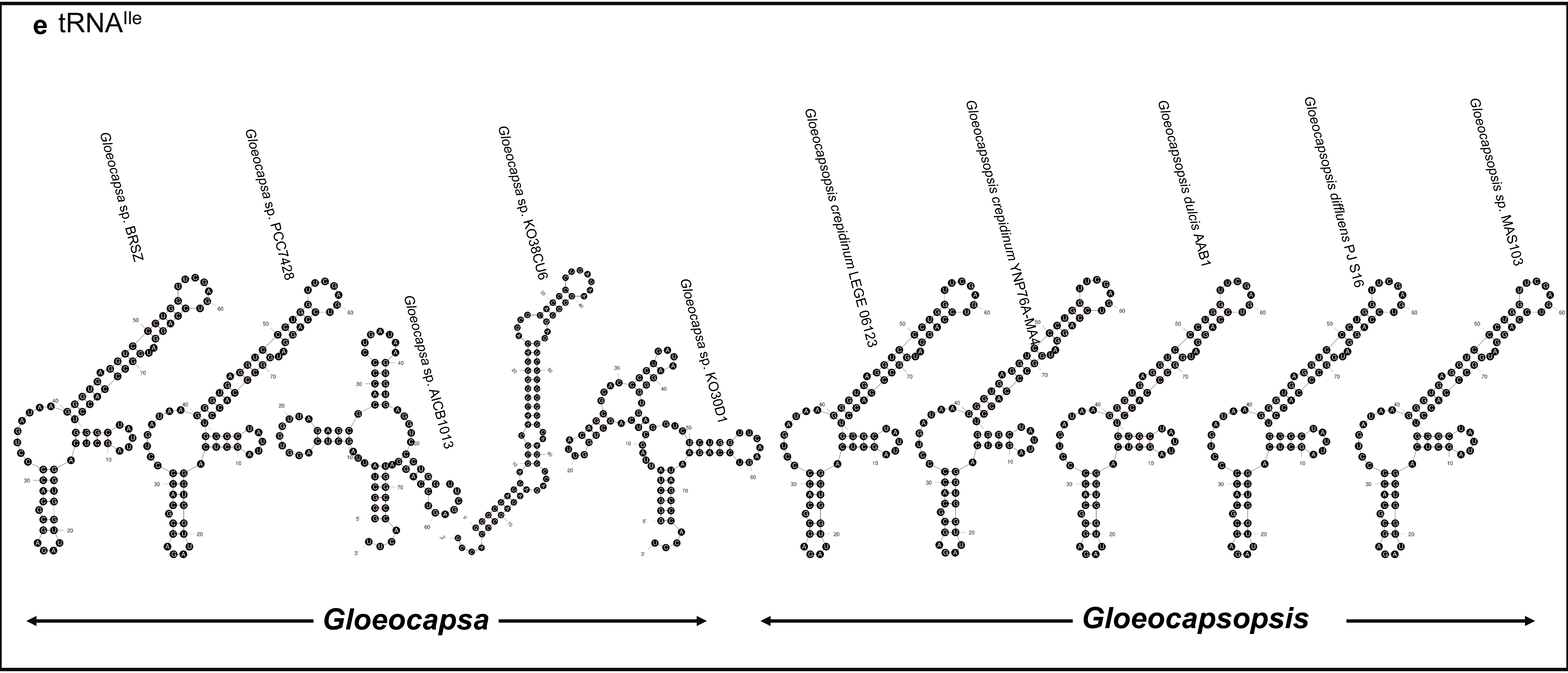
e tRNA^{Ile}
Gloeocapsa sp. BRSZ
Gloeocapsa sp. PCC7428
Gloeocapsa sp. AICB1013
Gloeocapsa sp. KO38CU6
Gloeocapsa sp. KO30D1
Gloeocapsopsis crepidinum LEGE 06123
Gloeocapsopsis crepidinum YNP76A-MA4
Gloeocapsopsis dulcis AAB1
Gloeocapsopsis diffluens PJ S16
Gloeocapsopsis sp. MAS103
Gloeocapsa
Gloeocapsopsis

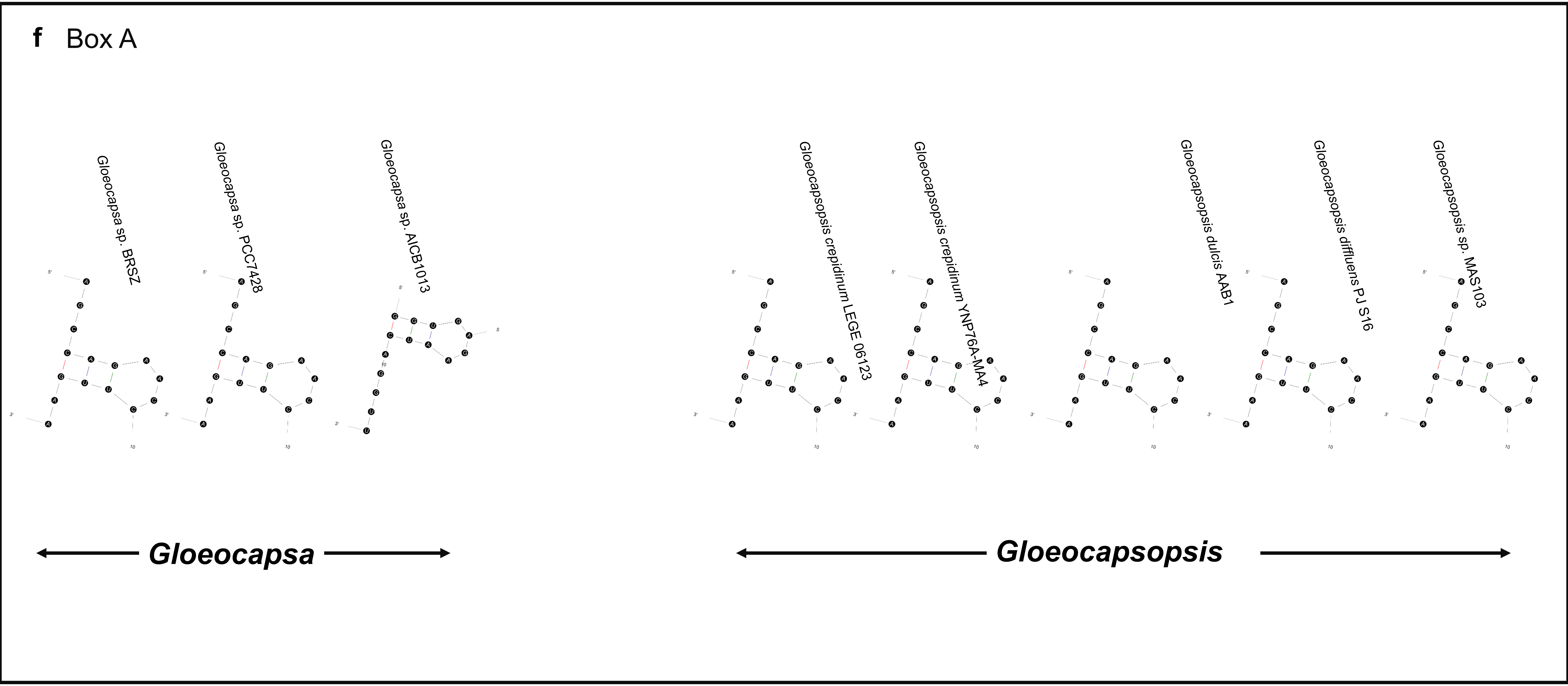
f
Box A
Gloeocapsa sp. BRSZ
Gloeocapsa sp. PCC7428
Gloeocapsa sp. AICB1013
Gloeocapsopsis crepidinum LEGE 06123
Gloeocapsopsis crepidinum YNP76A-MA4
Gloeocapsopsis dulcis AAB1
Gloeocapsopsis diffluens PJ S16
Gloeocapsopsis sp. MAS103
Gloeocapsa
Gloeocapsopsis

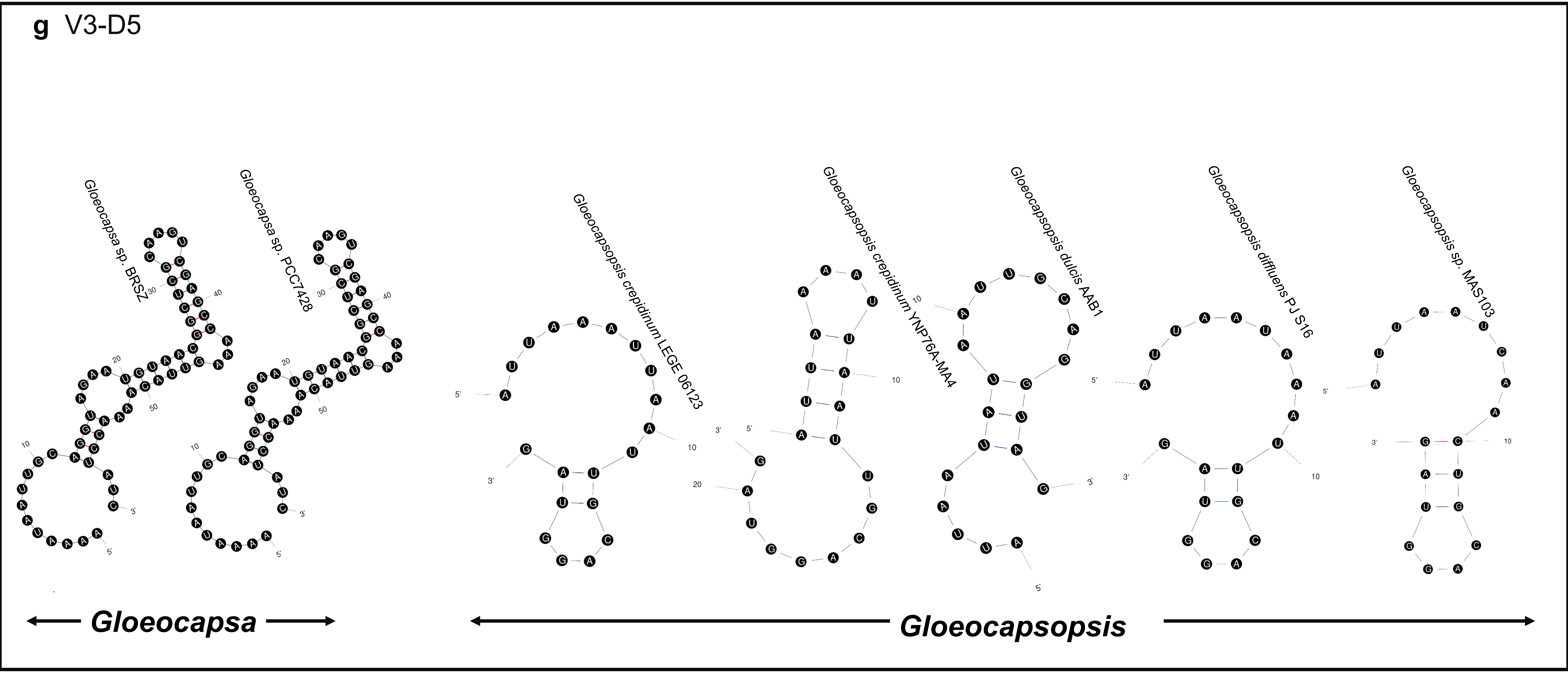
g V3-D5
Gloeocapsa sp. BRSZ
Gloeocapsa sp. PCC7428
Gloeocapsopsis crepidinum LEGE 06123
Gloeocapsopsis crepidinum YNP76A-MA4
Gloeocapsopsis dulcis AAB1
Gloeocapsopsis diffluens PJ S16
Gloeocapsopsis sp. MAS103
Gloeocapsa
Gloeocapsopsis
