# Supplementary material for: Genotypic and molecular characterization of a moderately thermophilic cyanobacterium, Gloeocapsa sp. strain BRSZ
Source: Eng Microbiol. 2025 Aug 5;5(3):100226. doi: 10.1016/j.engmic.2025.100226 (PMC12967839; doi:10.1016/j.engmic.2025.100226)
Supplement: Supplementary file 2 [file mmc2.pdf]

Table S1 The strains used in 16s rRNA phylogenetic analysis

No	Strain	Order	Family	GenBank Accession No.	Nucleotide region	Nucleotide length	% nucleotide similarity to strain BRSZ
1	*Gloeocapsa* sp. BRSZ	Chroococcales	Chroococcaceae	PP907067.1	1-1479	1479 bp	-
2	*Gloeocapsa* sp. PCC 7428	Chroococcales	Chroococcaceae	CP003646.1	4787969-4789447	1479 bp	98.85
3	*Gloeocapsa* sp. HG2	Chroococcales	Chroococcaceae	KY172947.1	1-1479	1479 bp	98.17
4	*Gloeocapsopsis* sp. MAS103	Chroococcales	Chroococcaceae	MZ677402.1	1-1478	1478 bp	97.02
5	*Gloeocapsopsis dulcis* AAB1	Chroococcales	Chroococcaceae	KU847399.1	229-1706	1478 bp	96.96
6	*Gloeocapsopsis crepidinum* strain LEGE 06123	Chroococcales	Chroococcaceae	NR_172660.2	1-1478	1478 bp	96.48
7	*Gloeocapsa quaternata* SERB 28	Chroococcales	Chroococcaceae	KM982577.1	1-1476	1476 bp	94.99
8	*Chroococcidiopsis* sp. CCMEE 010	Chroococcidiopsidales	Chroococcidiopsidaceae	OR413562.1	5-1481	1477 bp	92.21
9	*Chroococcidiopsis* sp. MAS97	Chroococcidiopsidales	Chroococcidiopsidaceae	MZ677393.1	1-1477	1477 bp	91.94
10	*Aliterella antarctica* strain CENA408	Chroococcidiopsidales	Aliterellaceae	NR_151904.1	1-1480	1480 bp	91.35
11	*Funiculus sociatus* LSB16 clone 1	Coleofasciculales	Coleofasciculaceae	MW403950.1	1-1479	1479 bp	91.14
12	*Nostoc calcicola* TSZ 2203	Nostocales	Nostocaceae	OQ627023.1	8-1484	1477 bp	91.06
13	*Calothrix* sp. NIES-2099	Nostocales	Calotrichaceae	LC455619.1	5-1481	1477 bp	91.00
14	*Tolypothrix* tenuis PCC 7101	Nostocales	Tolypothrichaceae	AP018248.1	1640366-1641842	1477 bp	91.00
15	*Desmonostoc* sp. CCIBT 3489	Nostocales	Nostocaceae	KX638490.1	1-1477	1477 bp	90.93
16	*Desmonostoc* sp. UHCC0398	Nostocales	Nostocaceae	ON693864.1	5-1481	1477 bp	90.93
17	*Desmonostoc* sp. CCM-UFV029	Nostocales	Nostocaceae	OP035734.1	1-1477	1477 bp	90.86
18	*Cylindrospermum catenatum* CCALA 999	Nostocales	Nostocaceae	KF052615.1	179-1654	1476 bp	90.85
19	*Desmonostoc* sp. CCM-UFV070	Nostocales	Nostocaceae	MG563381.2	1-1479	1479 bp	90.80
20	*Anabaena flos-aquae* strain UTEX LB2557	Nostocales	Aphanizomenonaceae	DQ234825.1	1-1477	1477 bp	90.79
21	*Tolypothrix* sp. PCC 7712	Nostocales	Tolypothrichaceae	CP063785.1	2408558-2410034	1477 bp	90.72
22	*Desmonostoc* sp. CCM-UFV083	Nostocales	Nostocaceae	OP035740.1	1-1477	1477 bp	90.66
23	*Tolypothrix* sp. PCC 7910	Nostocales	Tolypothrichaceae	CP050440.1	491138-492612	1475 bp	90.64
24	*Crassifilum sonorensis* strain SON62	Coleofasciculales	Coleofasciculaceae	NR_176555.1	5-1483	1479 bp	90.60
25	*Anabaena flos-aquae* strain UTEX LB2338	Nostocales	Aphanizomenonaceae	DQ234823.1	1-1477	1477 bp	90.59

No	Strain	Order	Family	GenBank Accession No.	Nucleotide region	Nucleotide length	% nucleotide similarity to strain BRSZ
26	*Trichormus variabilis* NIES-23	Nostocales	Nostocaceae	AP018216.1	483419-484895	1477 bp	90.52
27	*Anabaena* sp. 0830-A	Nostocales	Nostocaceae	AB936775.1	1-1477	1477 bp	90.52
28	*Nostoc* sp. PCC 7120	Nostocales	Nostocaceae	BA000019.2	2375741-2377217	1477 bp	90.52
29	*Microcoleus steenstrupii* LSB38 clone 2	Oscillatoriales	Microcoleaceae	MW403948.1	1-1479	1479 bp	90.47
30	*Microcoleus steenstrupii* LSB78 clone 1	Oscillatoriales	Microcoleaceae	MW403946.1	1-1479	1479 bp	90.47
31	*Goleter* sp. CHAB TP201702.1 clone 01	Nostocales	Nostocaceae	MT488126.1	1-1477	1477 bp	90.45
32	*Funiculus sociatus* SIK29 clone 1	Coleofasciculales	Coleofasciculaceae	MZ677331.1	1-1480	1480 bp	90.40
33	*Desmonostoc* sp. CCM-UFV082	Nostocales	Nostocaceae	OP035739.1	1-1478	1478 bp	90.39
34	*Cylindrospermum stagnale* PCC 7417	Nostocales	Nostocaceae	CP003642.1	3741618-3743094	1477 bp	90.39
35	*Aulosira laxa* NIES-50	Nostocales	Fortieaceae	KJ920353.1	186-1663	1478 bp	90.32
36	*Goleter* sp. CHAB TP201823.8	Nostocales	Nostocaceae	MT488273.1	2-1478	1477 bp	90.32
37	*Anabaena variabilis* ATCC 29413	Nostocales	Nostocaceae	CP000117.1	1002924-1004400	1477 bp	90.32
38	*Nostoc* sp. UAM 307	Nostocales	Nostocaceae	HM623782.1	1-1477	1477 bp	90.25
39	*Amazonocrinis nigriterrae* strain CENA67	Nostocales	Nostocaceae	NR_172622.1	1-1477	1477 bp	90.25
40	*Wilmottia stricta* 31PC	Coleofasciculales	Wilmottiaceae	KY288996.1	1-1477	1477 bp	90.25
41	*Anabaena variabilis* strain KCTC AG10273	Nostocales	Nostocaceae	DQ234828.1	1-1477	1477 bp	90.25
42	*Jaaginema* sp. CHAB TP201722.1	Synechococcales	-	MT488188.1	1-1480	1480 bp	90.20
43	*Jaaginema* sp. CHAB TP201722.4	Synechococcales	-	MT488193.1	1-1480	1480 bp	90.13
44	*Microcoleus* sp. ACSSI 305	Oscillatoriales	Microcoleaceae	MT425931.1	2-1479	1478 bp	90.12
45	*Chroococcidiopsis* sp. MAR110 clone 5	Chroococcidiopsidales	Chroococcidiopsidaceae	MZ677389.1	1-1479	1479 bp	90.06
46	*Anabaenopsis elenkinii* CCIBt3563	Nostocales	Aphanizomenonaceae	CP063311.1	1955053-1956529	1477 bp	90.05
47	*Scytonema hyalinum* MAR120 clone 1	Nostocales	Scytonemataceae	MZ677387.1	1-1475	1475 bp	90.03
48	*Desmonostoc* sp. CCM-UFV003	Nostocales	Nostocaceae	OP035723.1	1-1479	1479 bp	89.99
49	*Nostoc calcicola* AM50C	Nostocales	Nostocaceae	MG641901.1	1-1478	1478 bp	89.99
50	*Trichormus* sp. SBC124 clone 2	Nostocales	Nostocaceae	MW403966.1	1-1477	1477 bp	89.98
51	*Scytonema hyalinum* Mon66 clone C	Nostocales	Scytonemataceae	MK478708.1	1-1475	1475 bp	89.97
52	*Ramsaria avicennae* SM S12C	Coleofasciculales	Coleofasciculaceae	MF348316.1	11-1490	1480 bp	89.93

No	Strain	Order	Family	GenBank Accession No.	Nucleotide region	Nucleotide length	% nucleotide similarity to strain BRSZ
53	*Goleter* sp. CHAB TP201821.1 clone 03	Nostocales	Nostocaceae	MT488262.1	2-1478	1477 bp	89.91
54	*Wilmottia murrayi* 29PC	Coleofasciculales	Wilmottiaceae	KY288994.1	1-1477	1477 bp	89.91
55	*Lyngbya* sp. CHAB TP201718.1 clone 05	Oscillatoriales	Oscillatoriophycideae	MT488176.1	1-1479	1479 bp	89.86
56	*Calothrix* sp. PCC 7507	Nostocales	Calotrichaceae	CP003943.1	1913557-1915036	1480 bp	89.86
57	*Pycnacronema brasiliensis* 45PC	Coleofasciculales	Coleofasciculaceae	MF581661.1	1-1479	1479 bp	89.86
58	*Wilmottia murrayi* 27PC	Coleofasciculales	Wilmottiaceae	KY288992.1	1-1477	1477 bp	89.84
59	*Aphanizomenon ovalisporum* ILC-164	Nostocales	Aphanizomenonaceae	JF768744.1	8-1482	1475 bp	89.83
60	*Wilmottia koreana* FBCC-A812	Coleofasciculales	Wilmottiaceae	MN473879.1	4-1483	1480 bp	89.79
61	*Tolypothrix distorta* CAU1	Nostocales	Tolypothrichaceae	MG641913.1	1-1479	1479 bp	89.79
62	*Wilmottia koreana* strain FBCC-A812	Coleofasciculales	Wilmottiaceae	NR_172594.1	4-1483	1480 bp	89.79
63	*Pycnacronema brasiliensis* 44PC	Coleofasciculales	Coleofasciculaceae	MF581660.1	1-1479	1479 bp	89.79
64	*Desmonostoc* sp. CCM-UFV013	Nostocales	Nostocaceae	OP035729.1	1-1478	1478 bp	89.78
65	*Tolypothrix distorta* LSB87 clone 4	Nostocales	Tolypothrichaceae	MW403965.1	1-1478	1478 bp	89.78
66	*Sinocapsa zengkensis* CHAB6751	Chroococcidiopsidales	Sinocapsaceae	MG282255.1	2-1478	1477 bp	89.78
67	*Wilmottia murrayi* 28PC	Coleofasciculales	Wilmottiaceae	KY288993.1	1-1477	1477 bp	89.78
68	*Scytonema hyalinum* AM54A	Nostocales	Scytonemataceae	MG641905.1	1-1474	1474 bp	89.76
69	*Scytonema hyalinum* MAS93	Nostocales	Scytonemataceae	MZ677380.1	1-1474	1474 bp	89.76
70	*Scytonema hyalinum* Mon63 clone A	Nostocales	Scytonemataceae	MK478705.1	1-1473	1473 bp	89.75
71	*Desmonostoc* sp. CCM-UFV005	Nostocales	Nostocaceae	OP035725.1	1-1480	1480 bp	89.72
72	*Nodularia spumigena* UHCC 0039	Nostocales	Aphanizomenonaceae	CP020114.1	4209284-4210761	1478 bp	89.72
73	*Nodularia* sp. CHAB TP201507 clone 01	Nostocales	Aphanizomenonaceae	MT488087.1	1-1477	1477 bp	89.71
74	*Komarekiella atlantica* strain JM-1001-clone1	Nostocales	Nostocaceae	OR758489.1	2-1478	1477 bp	89.71
75	*Microcoleus steenstrupii* SIK77 clone 3	Oscillatoriales	Microcoleaceae	MZ677335.1	1-1480	1480 bp	89.59
76	*Phormidium murrayi* Ant-Ph58	Coleofasciculales	Wilmottiaceae	DQ493872.1	8-1485	1478 bp	89.58
77	*Crustifilum hispaliculae* strain CHAB7535-4	Coleofasciculales	Coleofasciculaceae	PP703005.1	2-1482	1481 bp	89.52
78	*Tolypothrix distorta* CAU13	Nostocales	Tolypothrichaceae	MG641916.1	1-1479	1479 bp	89.52
79	*Gloeotrichia echinulata* CP02	Nostocales	Gloeotrichiaceae	CP051187.1	1856876-1858350	1475 bp	89.49

No	Strain	Order	Family	GenBank Accession No.	Nucleotide region	Nucleotide length	% nucleotide similarity to strain BRSZ
80	*Microcoleus steenstrupii* SIK64 clone 7	Oscillatoriales	Microcoleaceae	MZ677334.1	1-1480	1480 bp	89.45
81	*Microcoleus steenstrupii* SIK79 clone 4	Oscillatoriales	Microcoleaceae	MZ677336.1	1-1480	1480 bp	89.45
82	*Tolypothrix distorta* Mon65 clone D	Nostocales	Tolypothrichaceae	MK478704.1	1-1479	1479 bp	89.45
83	*Nostoc* sp. HNBGU 006	Nostocales	Nostocaceae	OR237789.1	3-1480	1478 bp	89.38
84	*Phyllonema ansata* C694-M4 clone C694-M4 clone3	Nostocales	Rivulariaceae	KT936574.1	6-1481	1476 bp	89.36
85	*Potamolinea magna* strain 47PC	Oscillatoriales	Oscillatoriophycideae	NR_151862.1	1-1480	1480 bp	89.32
86	*Tolypothrix distorta* CANT1 clone 2	Nostocales	Tolypothrichaceae	MG641908.1	1-1479	1479 bp	89.32
87	*Potamolinea* sp. WZU166	Oscillatoriales	Oscillatoriophycideae	OM237450.1	7-1485	1479 bp	89.32
88	*Tolypothrix distorta* CANT3	Nostocales	Tolypothrichaceae	MG641909.1	1-1479	1479 bp	89.25
89	*Hassallia* sp. UB1-KK1 clone 3	Nostocales	Tolypothrichaceae	KF934132.1	162-1640	1479 bp	89.18
90	*Tolypothrix distorta* CAU14 clone 2	Nostocales	Tolypothrichaceae	MG641918.1	1-1478	1478 bp	89.17
91	*Nostoc azollae* 0708	Nostocales	Nostocaceae	CP002059.1	830919-832396	1478 bp	89.11
92	*Microcoleus steenstrupii* LSB38 clone 3	Oscillatoriales	Microcoleaceae	MW403949.1	1-1487	1487 bp	88.37
93	*Microcoleus steenstrupii* SBC109 clone 1	Oscillatoriales	Microcoleaceae	MW403939.1	1-1480	1480 bp	87.96
94	*Gloeobacter violaceus* PCC 7421	Gloeobacterales	Gloeobacteraceae	NR_074282.1	1-1485	1485 bp	85.06

Table S2 The length summary of regions within 16S-23S rRNA ITS of the strain BRSZ and 9 representative strains

No	Strain	GenBank Accession No.	Nucleotide region	% nucleotide similarity to strain BRSZ	Nucleotide length (bp)										
					Full ITS	D1-D1'	D2	D3	D2-D3	$tRNA^{Ile}$	V2	Box B	Box A	D4	V3-D5
1	*Gloeocapsa* sp. BRSZ	-	-	-	485	61	10	5	21	77	50	42	15	8	60
2	*Gloeocapsa sp.* PCC7428	CP003646.1	4789448-4789951	81.03	504	61	10	5	21	77	59	42	15	8	60
3	*Gloeocapsopsis crepidinum* LEGE 06123	FJ589716.1	1479-1886	68.14	408	61	10	5	21	77	52	41	15	8	20
4	*Gloeocapsopsis crepidinum* YNP 76A-MA4	OR259162.1	1161-1567	67.08	407	61	10	5	21	77	49	41	15	8	21
5	*Gloeocapsopsis dulcis* AAB1	KU847399.1	1707-2124	66.99	418	58	10	5	21	77	64	41	15	8	20
6	*Gloeocapsopsis diffluens* PJ-S16	MT986030.1	1456-1872	62.59	417	66	10	5	22	77	54	44	15	8	19
7	*Gloeocapsopsis* sp. MAS103	MZ677402.1	1479-1913	56.09	435	72	10	5	22	77	58	44	15	8	19
8	*Gloeocapsa* sp. KO38CU6	AB067575.1	1459-1851	36.39	393	98	10	5	21	77	45	31	9	-	-
9	*Gloeocapsa* sp. KO30D1	AB067579.1	1459-1851	38.93	393	98	10	5	21	77	45	31	9	-	-
10	*Gloeocapsa* sp. AICB1013	KJ746508.1	1414-1815	43.53	402	66	10	5	20	77	54	36	15	8	5

Table S3 Pairwise distance comparison calculated from the 16S-23S rRNA ITS sequence of the strain BRSZ and 9 representative strains

**Strain**	***Gloeocapsa* sp. BRSZ**	***Gloeocapsa*_sp. PCC7428**	***Gloeocapsopsis crepidinum* LEGE 06123**	***Gloeocapsopsis crepidinum* YNP 76A-MA4**	***Gloeocapsopsi dulcis* AAB1**	***Gloeocapsopsis diffluens* PJ-S16**	***Gloeocapsopsis* sp. MAS103**	***Gloeocapsa* sp. KO38CU6**	***Gloeocapsa* sp. KO30D1**	***Gloeocapsa* sp. AICB1013**
	**(1)**	**(2)**	**(3)**	**(4)**	**(5)**	**(6)**	**(7)**	**(8)**	**(9)**	**(10)**
**(1)**										
**(2)**	0.119									
**(3)**	0.215	0.204								
**(4)**	0.213	0.226	0.057							
**(5)**	0.222	0.236	0.206	0.201						
**(6)**	0.218	0.241	0.201	0.200	0.246					
**(7)**	0.204	0.251	0.234	0.242	0.606	0.149				
**(8)**	0.637	0.668	0.584	0.625	0.648	0.621	0.630			
**(9)**	0.621	0.651	0.585	0.626	0.640	0.613	0.605	0.014		
**(10)**	0.586	0.628	0.544	0.544	0.606	0.648	0.606	0.359	0.359	

Table S4 Comparative genome assembly metrics including assembly statistics and annotation details of *Gloeocapsa* sp. strain BRSZ and other two, PCC 7428 and PCC 73106

**Taxon**	***Gloeocapsa* sp. strain BRSZ**	***Gloeocapsa* sp. PCC 7428**	***Gloeocapsa* sp. PCC 73106**
**Genome assembly ID**	ASM4032831v1	ASM31755v1	ASM33203v1
**NCBI RefSeq assembly ID**	GCF_040328315.1	GCF_000317555.1	GCF_000332035.1
**Submitted GenBank assembly ID**	GCA_040328315.1	GCA_000317555.1	GCA_000332035.1
**WGS project ID**	JBEGHC01	*None*	ALVY01
**BioProject ID**	PRJNA1120971	PRJNA158831	PRJNA159497
**BioSample ID**	SAMN41723176	SAMN02261347	SAMN02261353
**Assembly statistics (GenBank)**			
**Genome size**	6,082,118 bp	5,882,710 bp	4,025,114 bp
**Total ungapped length**	6,081,827 bp	5,882,710 bp	4,025,114 bp
**Number of scaffolds**	42	5	228
**Scaffold N50**	552,304 bp	5,431,448 bp	35,959 bp
**Scaffold L50**	5	1	35
**Number of contigs**	45	5	228
**Contig N50**	543,782 bp	5,431,448 bp	35,959 bp
**Contig L50**	5	1	35
**GC percent**	43.5	43.5	41
**Genome coverage**	418x	30x	30x
**Assembly level**	Scaffold	Complete Genome	Contig
**Annotation details (GenBank)**			
**Provider**	NCBI	JGI	JGI
**Name**	NCBI Prokaryotic Genome Annotation Pipeline (PGAP)	Annotation submitted by JGI	Annotation submitted by JGI
**Genes**	5,585	5,304	4,150
**Protein-coding**	5,456	5,011	4,087

Table S5 the BRSZ-specific genes and proteins (stress tolerance, heterotrophy, and secondary metabolites)

No	Description	GenBank Accession No.	% Protein similarity	Strain
	**BRSZ-specific gene**			
1	Glycoside hydrolase family 3 (Thermostable beta-glucosidase B)	MES1022003.1	74.36	*Cyanosarcina radialis* HA8281-LM2
2	Uma2 family endonuclease	MES1022004.1	99.00	*Chroococcidiopsis* sp. CCMEE 29
3	Alpha-amylase family glycosyl hydrolase (Trehalose synthase)	MES1026485.1	94.94	*Gloeocapsopsis crepidinum*
4	GMC family oxidoreductase	MES1026245.1	98.46	Cyanobacteriota bacterium
5	Molecular chaperone DnaK	MES1025500.1	99.37	*Chroogloeocystis siderophila*
6	Molecular chaperone DnaJ	MES1025741.1	94.47	*Chroogloeocystis siderophila*
7	Co-chaperone GroES	MES1026386.1	99.03	*Gloeocapsopsis*
8	Glutathione S-transferase family protein	MES1021644.1	83.93	*Chroococcidiopsis* sp. CCMEE 29
9	Glutathione S-transferase family protein	MES1026383.1	91.21	*Gloeocapsopsis crepidinum*
	**BRSZ-proteins related to heterotrophy**			
1	sodium:solute symporter	MES1022636.1	99.63	*Gloeocapsa* sp. PCC 7428
2	MFS transporter	MES1023879.1	98.46	*Gloeocapsa* sp. PCC 7428
3	SulP family inorganic anion transporter	MES1023906.1	98.10	*Gloeocapsa* sp. PCC 7428
4	DNA/RNA nuclease SfsA	MES1026153.1	96.60	*Gloeocapsa* sp. PCC 7428
5	glucokinase	MES1024786.1	99.13	*Gloeocapsa* sp. PCC 7428
6	ROK family protein	MES1025310.1	99.34	*Gloeocapsa* sp. PCC 7428
7	carbohydrate kinase	MES1023907.1	98.45	*Gloeocapsa* sp. PCC 7428
8	type I phosphomannose isomerase catalytic subunit	MES1025485.1	96.34	*Gloeocapsa* sp. PCC 7428
9	xylulokinase	MES1025486.1	97.51	*Gloeocapsa* sp. PCC 7428
10	xylulokinase	MES1025490.1	98.18	*Gloeocapsa* sp. PCC 7428
11	glucose-6-phosphate isomerase	MES1026155.1	98.48	*Gloeocapsa* sp. PCC 7428
	**Secondary metabolite (predicted by antiSMASH)**	**Number of proteins with BLAST hit**		
1	Nostophycin	**7 proteins:** ResB-like protein, ABC transporter, GTP cyclohydrolase, putative RNA ligase, NpnA, NpnB, NpnC		
2	Minutissamide	**10 proteins:** PuwA, PuwI, PuwJ, PuwB, PuwC, PuwD, PuwK, PuwE, PuwF-G, PuwH		
3	Puwainaphycin	**7 proteins:** PuwA, PuwB, PuwC, PuwD, PuwE, PuwF-G, PuwH		
4	Nostopeptolide	**7 proteins:** nosA, nosB, nosC, nosD, nosE, nosF, nosG		
5	Anabaenopeptin	**5 proteins:** AptA, AptB, AptC, AptD, AptE		
